# Bouncing microdroplets on hydrophobic surfaces

**DOI:** 10.1073/pnas.2507309122

**Published:** 2025-09-04

**Authors:** Jamie McLauchlan, Jim S. Walker, Vatsal Sanjay, Maziyar Jalaal, Jonathan P. Reid, Adam M. Squires, Anton Souslov

**Affiliations:** ^a^Department of Physics, University of Bath, Bath BA2 7AY, United Kingdom; ^b^School of Chemistry, University of Bristol, Bristol BS8 1TS, United Kingdom; ^c^CoMPhy Lab, Department of Physics, Durham University, Science Laboratories, Durham DH1 3LE, United Kingdom; ^d^Van der Waals-Zeeman Institute, Institute of Physics, University of Amsterdam, Amsterdam 1098XH, The Netherlands; ^e^Department of Chemistry, University of Bath, Bath BA2 7AX, United Kingdom; ^f^TCM Group, Cavendish Laboratory, University of Cambridge, Cambridge CB3 0US, United Kingdom

**Keywords:** droplet, microfluidics, aerosols, bouncing, deposition

## Abstract

Intuitively, slow droplets stick to a surface and faster droplets splash or bounce. However, recent work suggests that on nonwetting surfaces, whether microdroplets stick or bounce depends only on their size and fluid properties, but not on the incoming velocity. Here, we show using theory and experiments that even poorly wetting surfaces have a velocity-dependent criterion for bouncing of aqueous droplets, which is as high as 6 m/s for diameters of 30 to 50 μm on hydrophobic surfaces such as Teflon. We quantify this criterion by analyzing the interplay of dissipation, surface adhesion, and incoming kinetic energy, and describe a wealth of associated phenomena, including air bubbles and satellite droplets. Our results on inertial microdroplets elucidate fundamental processes crucial to aerosol science and technology.

## Significance

Microdroplets play a critical role in understanding disease transmission, industrial processes, and natural phenomena. We focus on microdroplet bouncing, which has been less explored compared to impingement of larger-scale drops. Using experiments and theory, we find a fundamental criterion that predicts whether a microdroplet will stick or bounce off a hydrophobic surface based on its incoming velocity. Our finding presents a fundamental limit to the deposition of fast-moving microdroplets and lays the groundwork for advances in aerosol and microfluidic technologies that leverage these dynamics.

We breathe out small droplets and aerosols that splash, bounce, or stick onto surfaces, leading to contamination and disease transmission (1–7). Similar microdroplet phenomena are responsible for a variety of industrial (8), agricultural (9, 10), and environmental (11–15) processes. Droplet impingement has been extensively explored in millimetric droplets and is often motivated by printing applications (8, 16–22), yet remains unexplored for fast microdroplets on poorly wetting substrates.

Here, we ask a deceptively simple question: when does an aqueous microdroplet bounce? We contrast micron-size droplets with larger, millimeter-scale drops (17, 23–27). Large drops at small velocities stick to a substrate and spread to a flattened shape (8, 28–36), but fast millimeter-scale drops tend to splash (37–41), with the notable exception of bouncing on a thin air film (42–45) or bouncing off poorly wetting substrates (46, 47). In contrast, microdroplets with diameters of tens of microns occupy a different distinct regime due to their high surface-to-volume ratio, which creates a complex interplay between droplet dynamics, surface tension, and substrate adhesion. Splashing is rare, but sticking and bouncing are ubiquitous.

Substrate adhesion is a crucial factor dictating microdroplet bouncing, characterized by the contact angle θ via the Young–Dupré equation. For wetting surfaces, the droplets always stick and never bounce (48–51). In contrast, for superhydrophobic (i.e., nonwetting) surfaces, substrate adhesion plays no role and the droplets bounce unless all inertia is damped out by viscous dissipation (36, 52, 53). The dimensionless ratio of (dissipative) viscosity to (nondissipative) surface tension, the Ohnesorge number Oh, defines a simple criterion for microdroplets when surface adhesion is negligible: droplets bounce when Oh is less than a constant of order 1, independent of incoming velocity (46, 47). For water microdroplets at room temperature, the Ohnesorge number has a relatively low value, Oh≈0.02, which shows that the droplets are underdamped and that both substrate adhesion and dissipation in the substrate-adjacent boundary layer must play a critical role. Together, these results point toward a transition on hydrophobic substrates that depends on contact angle and velocity.

In this work, we find that the transition from sticking to bouncing in microdroplets occurs when the incoming kinetic energy overcomes both dissipation and substrate adhesion. We focus on experiments with large microdroplets (around 30 to 50μm in diameter) impinging on a hydrophobic Teflon surface with speeds of 1 to 10 m/s; these impacts occur over tens of microseconds and represent some of the fastest and smallest-scale droplet bouncing events ever experimentally captured in high-resolution detail. The mechanism of this stick-to-bounce transition can be understood using a simple underdamped ball-and-spring model, backed up by computational fluid dynamics that parallel our experimental results. These findings shed light on the previously unexplored interplay between inertia and adhesion crucial for applications from inkjet and 3D printing to sprays and aerosol resuspension.

## Experimental Stick-to-Bounce Transition

**Fig. 1.**
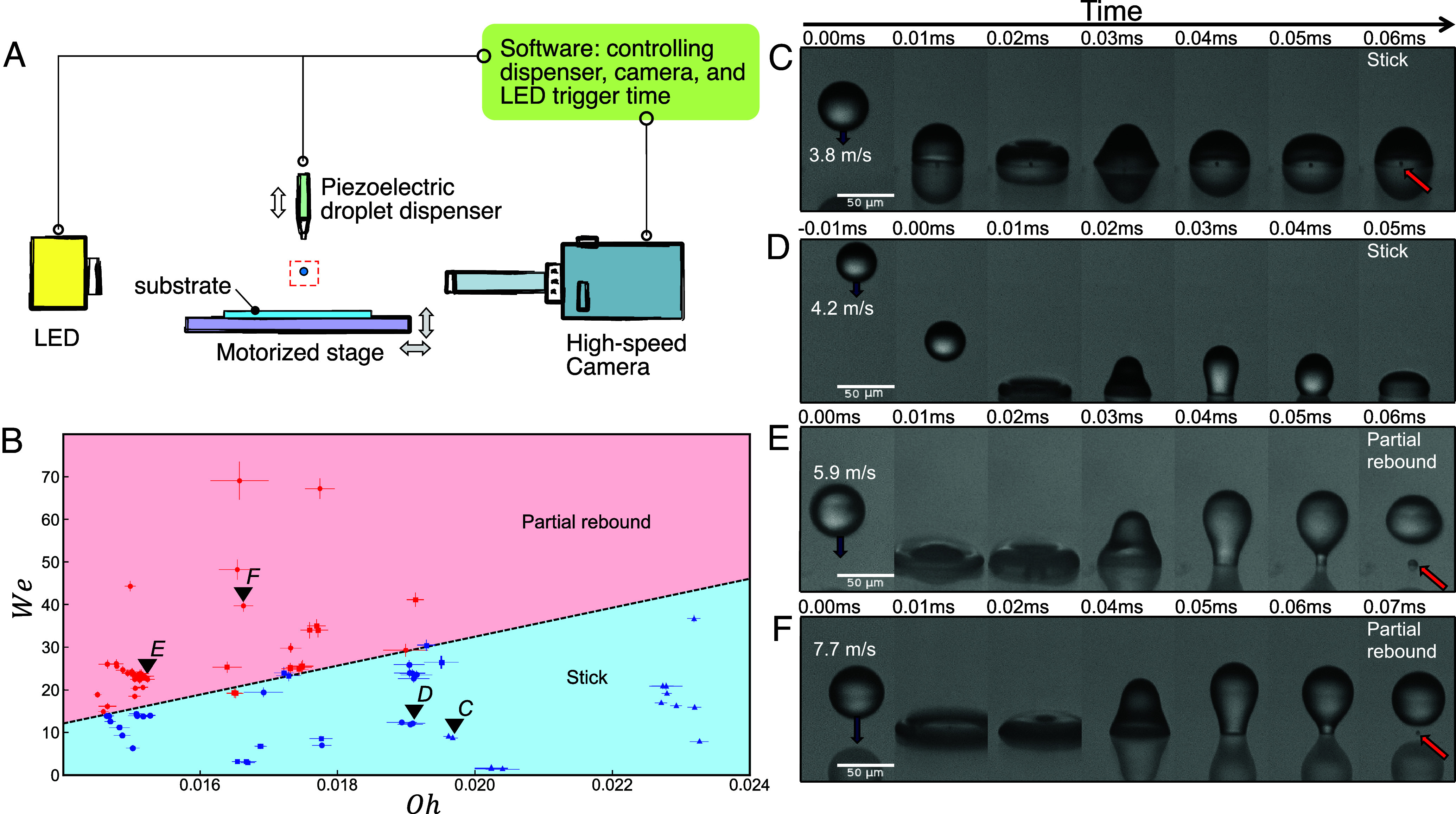
Microdroplet experiments. (*A*) Schematic of experimental setup, in which an aqueous droplet is dispensed and imaged on a high-speed camera. (*B*) Experimental results plotted within the droplet phase space of Weber We vs. Ohnesorge Oh numbers for a Teflon surface with a contact angle of 110°. For each experimental data point, represented by a circle (water), square (2.5%CaCl_2_), or triangle (5% glycerol), the outcome was classified as a sticking event [e.g., points labeled as (*C* and *D*) and shown in the corresponding subfigure] or a partial rebound [e.g., subfigures (*E* and *F*)]. Error bars denote measurement uncertainties. The transition line is a guide to the eye separating the experimental outcomes. (*C*–*F*) High-speed imaging of droplet impact outcomes: the recorded outcomes are water droplets on a Teflon surface with a static contact angle of 110°. (*C*) Sticking outcome, with an entrained air bubble shown by red arrow. Observed oscillations indicate that sticking occurs in the underdamped regime. (Oh,We)=(0.0197,8.80). (*D*) Sticking outcome, without an air bubble but with oscillations. (Oh,We)=(0.0191,12.0). (*E*) Partial rebound, with a small sessile droplet shown by red arrow. (Oh,We)=(0.0152,23.7). (*F*) Partial rebound, with a smaller sessile droplet (red arrow). (Oh,We)=(0.0166,39.8).

In our experiments, we impact water-based microdroplets onto a poorly wetting Teflon substrate and use a high-speed camera to classify the outcomes as either sticking or partially rebounding; see Fig. 1. In the experiments, each impact samples from a range of velocities u (between 1 and 10m/s) and droplet diameters D (between 30 and 50 μm), and we vary the dynamic viscosity μ (between $0.88\times 10^{-3}$ and $1.05\times 10^{-3}\text{Pa}\cdot \text{s}$) by varying temperature and glycerol/salt concentration, while keeping surface tension γ approximately constant; see *Materials and Methods*. Other relevant parameters, such as density ρ, and the surface-dependent static contact angle θ are also kept constant. Together, these parameters form a distinct region in the two-dimensional phase space spanned by the Weber number We, defined as

[1] $$\begin{array}{c} \mathrm{We}\equiv \frac{\rho Du^{2}}{\gamma }, \end{array}$$

and Ohnesorge number Oh,

[2] $$\begin{array}{c} \mathrm{Oh}\equiv \frac{\mu }{\sqrt{\rho \gamma D}}, \end{array}$$

where the denominator $\sqrt{\rho \gamma D}$ represents a combination of inertial and capillary effects and is independent of the impact velocity. For our experimental parameters, We varies between 1 and 70, and Oh varies between 0.014 and 0.024, which is much lower than in inkjet printers (8). The prominence of inertia is captured in the Reynolds number Re≡We1/2Oh−1, which ranges from 50 to 500 in our experiments. Unlike millimeter-scale droplets (46, 47) gravity does not play a role during impact, with a Bond number $\mathrm{Bo}\equiv \rho gD^{2}/\gamma \approx O\left(10^{-4}\right)$. Electrostatic charging effects (tens of femtocoulombs per droplet) do not noticeably alter the bouncing dynamics; however, they can influence the postbounce dynamics; see *SI Appendix* for details. We measure the static contact angle on our hydrophobic Teflon surface to be 110° with moderate hysteresis of 19° consistent with nm-scale surface roughness as measured by atomic force microscopy (AFM); see *SI Appendix*.

Slow microdroplets, with a relatively low *We*, adhere to the substrate after impact. Fig. 1 *C* and *D* illustrate how the droplet initially flattens and inertially spreads outward. The contact line advances at a large angle until the radial spread reaches a maximum. Capillary forces then drive the fluid to retract and the droplet performs several underdamped oscillations (*SI Appendix*, Fig. S3), in contrast to the previously explored overdamped regime (47). In some cases, the droplet encloses an air bubble, see Fig. 1*C*, because of the collapse of the thin film of air formed underneath the droplet during impingement. Others have reported contactless bouncing on this air film (42–45), but in our experiments, the air cushion is always unstable and instead bouncing is controlled by surface adhesion.

By contrast, fast microdroplets, with a relatively high *We*, perform a partial rebound while leaving behind a sessile remnant. In these cases, after the initial spreading and retraction, the droplet undergoes a necking instability and detaches from the surface; see Fig. 1 *E* and *F*. Our combined data in Fig. 1*B* shows the outcomes in (Oh,We) parameter space, with a line separating the sticking at high Oh and low We from the partial rebounds at low Oh and high We. The positive slope of this line notably contrasts to the case of a superhydrophobic surface, ref. 47, for which the (purely vertical) transition line Oh≈1 is independent of We and the incoming velocity. The effect of even a small surface adhesion is apparent: at small Oh, the droplet adheres to the hydrophobic surface and can stick even in the underdamped regime. However, at high incoming velocities (i.e., high We), the initial kinetic energy overcomes this surface adhesion and allows the droplet to escape. The small sessile droplet that stays on the surface provides additional evidence that, in this case, sticking results from surface adhesion.

## Numerical Simulations

This experimental phenomenology can be quantitatively captured in finite-element numerical simulations based on a few simple ingredients. Once calibrated, we use these simulations to explore parameter regimes (in terms of Oh, We, and contact angle θ) inaccessible in our experiments.

**Fig. 2.**
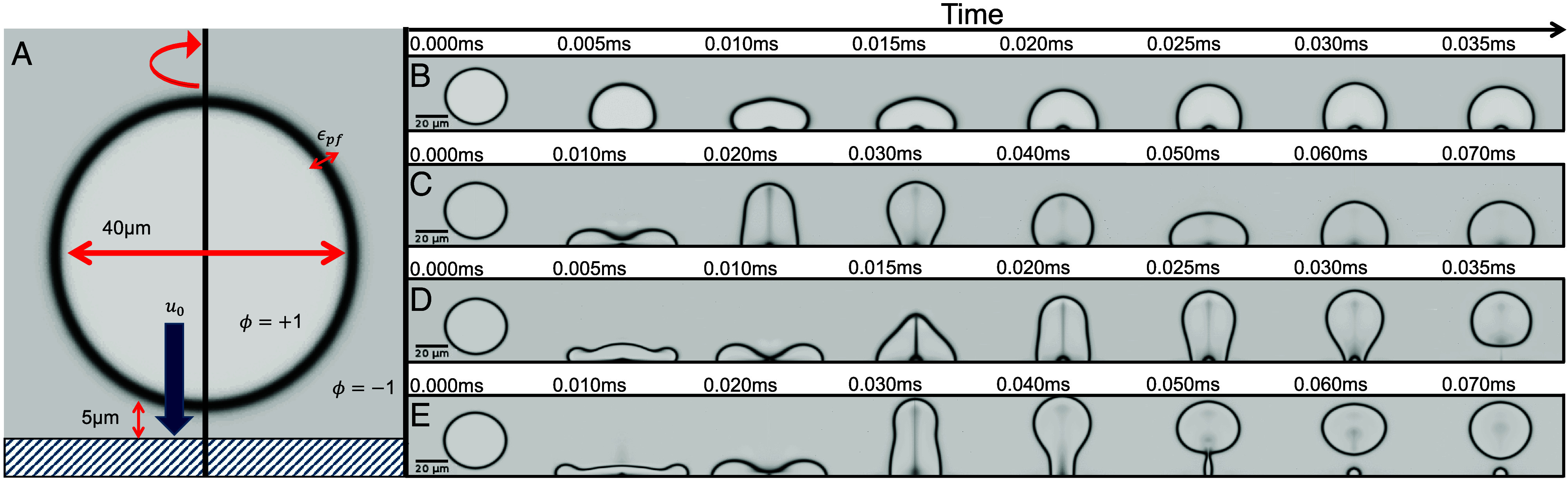
(*A*) Schematic of finite-element phase-field simulations for microdroplet bouncing. (*B*–*E*) Numerical simulations reproduce the experimentally observed outcomes: (*B*) Sticking with an air bubble and small oscillations, $\left(\mathrm{Oh},\mathrm{We},\theta \right)=\left(0.019,2,110^{{}^{\circ}}\right)$. (*C*) Sticking with a large maximum spread and large oscillations, $\left(\mathrm{Oh},\mathrm{We},\theta \right)=\left(0.028,56,110^{{}^{\circ}}\right)$. (*D*) Total rebound with a large maximum spread and an air bubble, $\left(\mathrm{Oh},\mathrm{We},\theta \right)=\left(0.019,27,110^{{}^{\circ}}\right)$. (*E*) Partial rebound with a large initial spread and a necking instability, $\left(\mathrm{Oh},\mathrm{We},\theta \right)=\left(0.011,42,100^{{}^{\circ}}\right)$.

We perform computational fluid dynamics using finite-element software COMSOL (54) Multiphysics in an axially symmetric geometry (Fig. 2*A*). We track the droplet–air interface using a phase-field variable ϕ which interpolates between −1 and +1, and which follows Cahn–Hilliard dynamics (55–57); see *Materials and Methods*. Although we use a finite-element solver, we check that the droplet volume is conserved by computing a surface integral of ϕ across the entire domain. In the simulations, we integrate the incompressible Navier–Stokes equations:

[3] $$\begin{array}{c} \rho \partial_{t}u+\rho \left(u\cdot \nabla \right)u=\nabla \cdot \sigma -\nabla p+F_{\mathrm{ST}}, \end{array}$$

where u(x,y) is the velocity field, p is the pressure, $F_{\mathrm{ST}}$ is the force due to surface tension, and σ is the viscous stress tensor. We implement incompressibility and a no-slip boundary condition for the substrate with a static contact angle at the contact line.

**Fig. 3.**
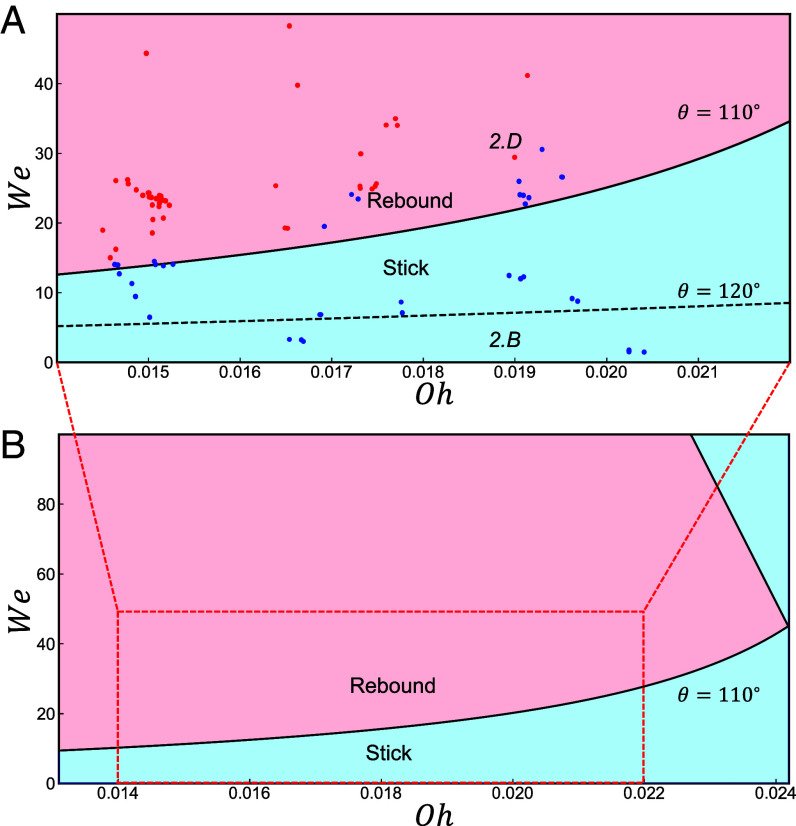
(*A*) Droplet outcome comparing numerical and experimental results, where the black line indicates the numerical transition from sticking (blue) to bouncing (red) for $\theta =110^{{}^{\circ}}$. The dotted line shows that the same transition for the larger contact angle $\theta =120^{{}^{\circ}}$ occurs at lower We (*SI Appendix*, Fig. S5 for numerical data points and *SI Appendix*, Fig. S6 for a rescaled plot in the Reynolds vs. Ohnesorge numbers phase space). The data points are the experimental results from Fig. 1. (*B*) A view of the (Oh,We) parameter space using simulations with a larger range of values and $\theta =110^{{}^{\circ}}$. For higher impact velocities (i.e., higher We), the droplets again begin to stick, showing the reentrant nature of the transition.

We show sample simulation results in Fig. 2*B*–*E*, which are broadly consistent with experiments. For low Weber number We and $\theta =110^{{}^{\circ}}$ (consistent with experiments on Teflon), the droplets stick to the substrate and can entrap an air bubble; see Fig. 2 *B* and *C*. At higher We, the air bubble remains, but the droplet rebounds, Fig. 2*D*. While the air bubble is not always present in experiments, its appearance in both simulations and some experimental sticking cases (e.g., Fig. 1*C*) suggests the simulations capture the qualitative entrapment behavior. As the bubble does not significantly influence the impact outcome, we consider it a noncritical feature. Combining all of the numerical outcomes, Fig. 3*A* shows the transition line from sticking to bouncing for two values of the contact angle.

Increasing the contact angle in the simulations above $\theta =110^{{}^{\circ}}$ lowers the We of the transition to sticking for a given Oh, with no sticking in this inertial regime when the contact angle approaches 180°. This confirms our intuition that the inertial effects encoded in *We* interplay against the surface adhesion encoded in *θ*: the larger the contact angle, the weaker the adhesion, and the less inertia is necessary to overcome it.

The simulations allow us to access a counterintuitive regime for We larger than in experiments, in which the droplets transition from bouncing back to sticking as their velocity is increased, Fig. 3*B*. This upper transition occurs because the droplet spreads more and therefore stores a larger amount of energy in the surface. In turn, the droplet then dissipates a larger fraction of its incoming kinetic energy (46) at larger incoming velocities. For larger values of the viscosity (i.e., larger *Oh*), this extra dissipation is sufficient to overcome the kinetic energy needed for a rebound (at sufficiently small contact angles θ). The intersection of these two re-entrant transitions leads to an overall maximum value of ${\mathrm{Oh}}_{\text{crit}}\approx 0.024$ above which only sticking outcomes are observed (for $\theta =110^{{}^{\circ}}$). Notably, this value is significantly smaller than 1, indicating that sticking still occurs in the inertial regime and for any incoming droplet velocity accessible in our experiments (i.e., any We). However, as the θ increases, so does ${\mathrm{Oh}}_{\text{crit}}$, reaching ${\mathrm{Oh}}_{\text{crit}}\approx 1$ in the superhydrophobic limit, where the sticking–bouncing transition becomes independent of We (47).

**Fig. 4.**
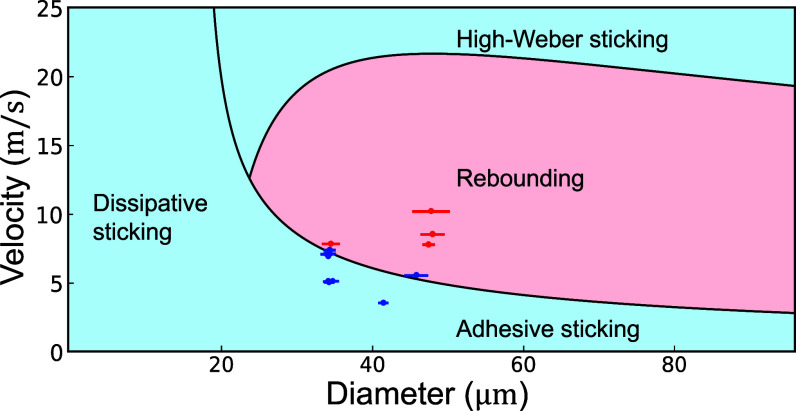
Rebounding and sticking in the phase space of Velocity vs. droplet Diameter for poorly wetting surface, $\theta =110^{{}^{\circ}}$, corresponding to the redimensionalized We−Oh axes in Fig. 3. We keep other parameters constant and plot experimental data for water on Teflon at room temperature as points (subset of Fig. 1 data recorded at the same viscosity and size) and finite-element simulations as lines. The graph indicates three distinct sticking mechanisms, with boundaries derived from the numerical results: dissipative sticking occurs for small droplets whose incoming velocity is fully dissipated by viscosity; adhesive sticking occurs when droplets are underdamped but cannot rebound due to surface adhesion; and high-velocity sticking occurs when kinetic energy is dissipated through larger maximum spreading at high impact speeds. See *SI Appendix*, Fig. S7, for a nondimensional label of these regions in combination with theory.

From the criterion Oh<0.024 for $\theta =110^{{}^{\circ}}$, we conclude that there is a universal size limit for aqueous droplets, below which bouncing does not occur for any velocity. We find that water droplets at room temperature cannot bounce from a surface with contact angle $\theta =110^{{}^{\circ}}$ if D<25μm as shown in Fig. 4, and this threshold decreases for more hydrophobic surfaces, for example D<10μm for $\theta =120^{{}^{\circ}}$. We conclude that aqueous aerosols with 10n m≲D≲1μm can only bounce off superhydrophobic surfaces with $\theta >150^{{}^{\circ}}$ and bouncing is suppressed for Oh≳1. Overall, bouncing for moderately hydrophobic surfaces occurs within a ’Goldilocks zone’ of moderately large We and moderately small Oh, where the droplet has sufficient incoming kinetic energy to overcome adhesive effects without excessive dissipation during the spreading and receding process. This zone expands in size with increasing contact angle until it reaches its maximum size at $180^{{}^{\circ}}$, where the boundary becomes Oh=1 in agreement with the previously understood superhydrophobic regime (46, 47).

The rebounds in Fig. 2*B*–*D* do not deposit a sessile droplet, which suggests that these simulations miss some of the experimental complexity, such as contact angle hysteresis. Nevertheless, partial rebounds can be simulated by changing the contact angle and other simulation parameters such as the fluid density; see Fig. 2*E*. However, the bouncing mechanism appears to be the same for both partial and total rebounds, and we proceed to formulate quantitative models that capture both cases.

## Energy Balance Criterion

The complex dynamics that govern the outcome of the droplet–surface interaction can be heuristically understood through the lens of energy conservation. A droplet will only rebound if the kinetic energy after lift-off is positive, $E_{k,f}>0$. This criterion can be restated as $E_{k,f}=E_{k,0}-E_{\gamma }-E_{\mu }>0$, where the initial kinetic energy $E_{k,0}$ decreases by the energy of the newly created droplet–surface contact $E_{\gamma }$ and the energy $E_{\mu }$ associated with viscous dissipation during the impact process. Phenomenologically, almost all dissipation occurs during the retraction process, so $E_{k,0}$ can be considered as the energy at maximum droplet spread. This kinetic energy contains the velocity dependence of the stick-to-bounce transition: $E_{k,0}\equiv \frac{1}{2}mu_{0}^{2}$, where $u_{0}$ is the incoming velocity of the droplet center-of-mass before surface contact. A heuristic criterion for the velocity $u_{0}$ at this transition is then:

[4] $$\begin{array}{c} \frac{1}{2}mu_{0}^{2}=E_{\gamma }+E_{\mu }. \end{array}$$

This expression does not explicitly include dissipative work during the spreading and retraction phases, which converts some kinetic energy to heat via fluid damping and pressure–volume work. These contributions are small in the regime considered but become more relevant at higher We.

The kinetic energy loss during a bouncing process, $E_{\gamma }+E_{\mu }$, can be quantified using simple scaling arguments. The surface energy scales as $E_{\gamma }=\pi \gamma D^{2}f\left(\theta \right)$, where $\pi \gamma D^{2}$ is the surface energy scale for a spherical surface, $\pi D^{2}$, and the contact angle θ encodes the difference between the air–droplet and the surface–droplet energies per unit area. A form for f(θ) is derived in the SI under the assumption that a small sessile droplet remains on the surface but will be left arbitrary here.

The energy $E_{\mu }$ dissipated by viscosity is more complex to quantify because it involves contributions from three different mechanisms: i) $E_{\mu ,3D}$ in the three-dimensional flows in the droplet bulk, ii) $E_{\mu ,2D}$ in the boundary layer associated with the contact area between the droplet and the surface (58), and iii) $E_{\mu ,1D}$ along the air–droplet–surface contact line. Based on inertial dynamics, dimensional analysis requires that both bulk and surface dissipation scale as (59)

[5] $$\begin{array}{c} E_{\mu ,3D}∼E_{\mu ,2D}∼\mu u_{0}^{2}Dt_{c}, \end{array}$$

in terms of the contact time $t_{c}$ (*SI Appendix*, Fig. S1), but with different scaling coefficients, so in combination $E_{\mu ,3D}+E_{\mu ,2D}=\kappa \mu u_{0}^{2}Dt_{c}$, where κ is a dimensionless proportionality constant. Although κ can itself depend on droplet parameters such as We and boundary layer thickness, we assume it to be a constant for simplicity.

Contact line dissipation is a distinct process dominated by the friction of the contact line moving along the surface and depinning from surface imperfections. The energy $E_{\mu ,1D}$ depends instead on the maximum spread diameter $D_{m}$, the surface tension, and the contact angle hysteresis $\Delta \cos\theta \equiv \cos\theta_{a}-\cos\theta_{r}$ via (60, 61):

[6] $$\begin{array}{c} E_{\mu ,1D}\approx D_{m}^{2}\gamma \;\Delta \cos\theta . \end{array}$$

**Fig. 5.**
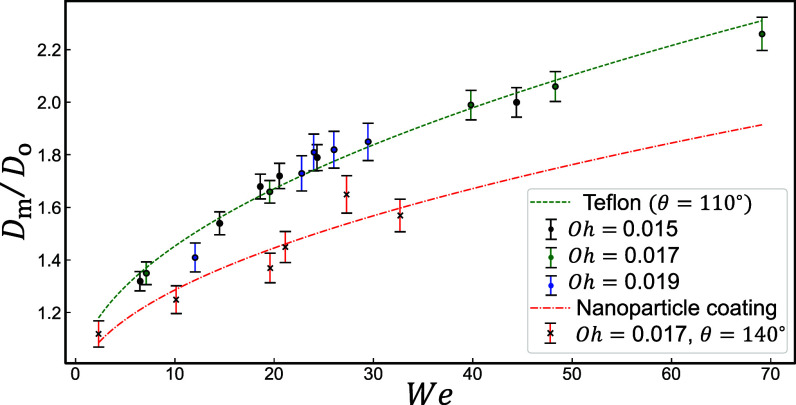
Experimental measurement of the diameter at maximum spread, $D_{\text{m}}$, normalized by droplet diameter D, for $\left(\mathrm{Oh},\theta \right)=\left(0.015,110^{{}^{\circ}}\right)$, $\left(0.017,110^{{}^{\circ}}\right)$, $\left(0.019,110^{{}^{\circ}}\right)$, and $\left(0.017,140^{{}^{\circ}}\right)$, for two different surfaces: Teflon and nanoparticle-coated glass. Both fits are consistent with theoretical prediction in Eq. **7** with different θ-dependent prefactors. These data show that the maximum spread is independent of Oh (*SI Appendix*, Fig. S2).

The maximum spread diameter $D_{m}$ is an important variable in droplet systems but its exact form has been widely debated in the literature (51, 62–67). We fit to a widely accepted general form:

[7] $$\begin{array}{c} \frac{D_{m}}{D}=g\left(\theta \right)\left(1+C{\mathrm{We}}^{1/2}\right), \end{array}$$

where C is a constant and g(θ) accounts for the wettability of the surface. This expression was previously derived in ref. 64 and has shown to hold true for a wide range of drop impacts (65, 67). In the narrow range of 10<We<100, another phenomenological expression, $D_{m}/D\approx {\mathrm{We}}^{1/4}$, is also commonly used (51, 62). From our experimental data, Fig. 5, we find that C≈0.14 and the spread is independent of Oh. Substituting Eq. **7** into Eq. **6**, we arrive at the scaling result for contact line dissipation, $E_{\mu ,1D}\approx D^{2}\gamma h\left(\theta ,\Delta \theta \right){\left(1+C{\mathrm{We}}^{1/2}\right)}^{2}$, where $h=g^{2}\left(\theta \right)\;\Delta \cos\theta$.

Combining these scaling laws, we find that the droplet will have sufficient kinetic energy to bounce if

[8] $$\begin{array}{c} E_{k,0}>\pi \gamma D^{2}f\left(\theta \right)+\kappa \mu u_{0}^{2}Dt_{c}+D^{2}\gamma h\left(\theta ,\Delta \theta \right){\left(1+C{\mathrm{We}}^{1/2}\right)}^{2}. \end{array}$$

Rescaling by $D^{2}\gamma$, we obtain the prediction for the stick-to-bounce transition (see *SI Appendix* for a complete derivation):

[9] $$\begin{array}{c} \mathrm{We}=\frac{f\left(\theta \right)\;+\;h\left(\theta ,\;\Delta \theta \right)\left(1\;+\;2C{\mathrm{We}}^{1/2}\right)}{1\;-\;\kappa \;\mathrm{Oh}\;-\;C^{2}h\left(\theta ,\;\Delta \theta \right)}. \end{array}$$

This is the most general expression, however excluding the $E_{\mu ,1D}$ hysteresis contributions, it simplifies to a more intuitive form that provides a good approximation for systems with low hysteresis:

[10] $$\begin{array}{c} \mathrm{We}=\frac{f(\theta )}{1-\kappa \mathrm{Oh}}. \end{array}$$

Eq. **10** interpolates between two extremes: i) the regime of small Oh (and small We) in this work, for which the stick-to-bounce transition is given by the linear relation We(Oh) and the slope of the line is determined by the contact angle, and ii) the asymptotic limit of the transition for large We, Oh≈1/κ, which recovers the velocity-independent transition predicted for superhydrophobic surfaces (47). For case (ii), the (constant) parameter κ captures how the threshold Oh is lowered by boundary-layer dissipation due to the interplay between wetting and droplet spreading. Overall, Eq. **10** matches well the observed phenomenology and provides quantitative confirmation for the energy-balance mechanism underlying droplet sticking and bouncing. The transitions from the numerical work in Fig. 4 can be interpreted within this framework, where case (i) corresponds to adhesive sticking, case (ii) to dissipative sticking, while high-velocity sticking falls outside the scope of this treatment; see *SI Appendix* for a dimensionless version of this figure.

**Fig. 6.**
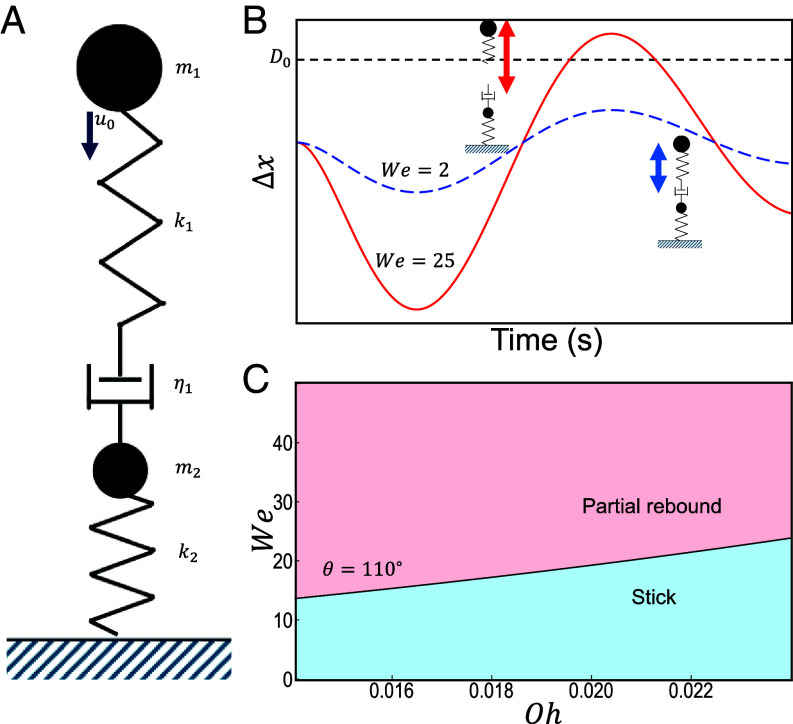
(*A*) An illustration of the simple model of two masses, two springs, and a damper to represent a microdroplet impact. (*B*) The extension Δx is plotted for two representative cases, one where Δx exceeds the bouncing threshold (red) and one where it does not (blue, with lower initial velocity). (*C*) We−Oh phase space for this model with (α,ζ,θ) = (50,1,110°) . This minimal model reproduces the simulation and experimental results (see *SI Appendix*, Fig. S7 for a further phase space).

The phenomenological argument for energy balance leads us to construct a simple ball-and-spring model that captures both bouncing and sticking, Fig. 6*A*. A large upper mass $m_{1}$ (representing the bulk of the droplet) is connected to a smaller mass via a spring and dashpot in series, with the smaller mass $m_{2}$ (representing the part of the droplet that makes contact with the surface) attached to the surface through a second spring. A damper represents the total dissipation in the system, the upper spring represents droplet surface tension, and the lower spring represents the adhesion between the surface and the fluid. Although the model simplifies the full impact physics, we retain only the essential features responsible for the stick-to-bounce transition. Effects such as contact angle hysteresis are neglected, while overall dissipation is captured through a calibrated damping term (*SI Appendix*). The particles first impact the surface until the springs are maximally compressed, corresponding to maximum droplet spreading. The energy stored in the springs corresponds to the energy in both the fluid–air and fluid–solid interfaces at maximum droplet spreading. As the springs relax from this compression, in the underdamped regime, some of the energy is dissipated by the dashpot, and the rest of the energy is converted into spring extension. Fig. 6*B* illustrates how we define a bouncing outcome in this model: the upper spring is considered to break above a threshold extension, during the first period of the spring oscillation.

Using dimensional rescaling of the spring model, the dynamics can be reexpressed in terms of the *We* and *Oh* numbers,

[11] $$\begin{array}{cc} m_{1}{\ddot{x}}_{1} & =-\frac{\zeta }{\mathrm{We}}\Delta x-\frac{\alpha \mathrm{Oh}}{\sqrt{\mathrm{We}}}\Delta \dot{x}\\ m_{2}{\ddot{x}}_{2} & =\frac{\zeta }{\mathrm{We}}\Delta x+\frac{\alpha \mathrm{Oh}}{\sqrt{\mathrm{We}}}\Delta \dot{x}-\left[1+\cos\left(\theta \right)\right]\zeta {\mathrm{We}}^{-1}x_{2}, \end{array}$$

where all of the quantities have been nondimensionalized, $m_{1,2}$ are the mass fractions of the two droplet parts, ζ is the spring constant (of order 1), α is the damping (of order 50), $x_{1,2}$ are the coordinates of the upper and lower particles, respectively, and $\Delta x=x_{1}-x_{2}$ is the extension of the upper spring (see *SI Appendix* for rescaling). Fig. 6*C* shows the stick-to-bounce transition using experimental values of We and Oh within this model, reproducing the transition line observed in simulations and experiments.

Overall, the ball-and-spring model translates the energy balance argument into the simplest dynamical model and shows the mechanism that relates surface adhesion to the velocity at which the transition is observed for a given Oh.

## Experiments on Nanoparticle-Coated Surface

**Fig. 7.**
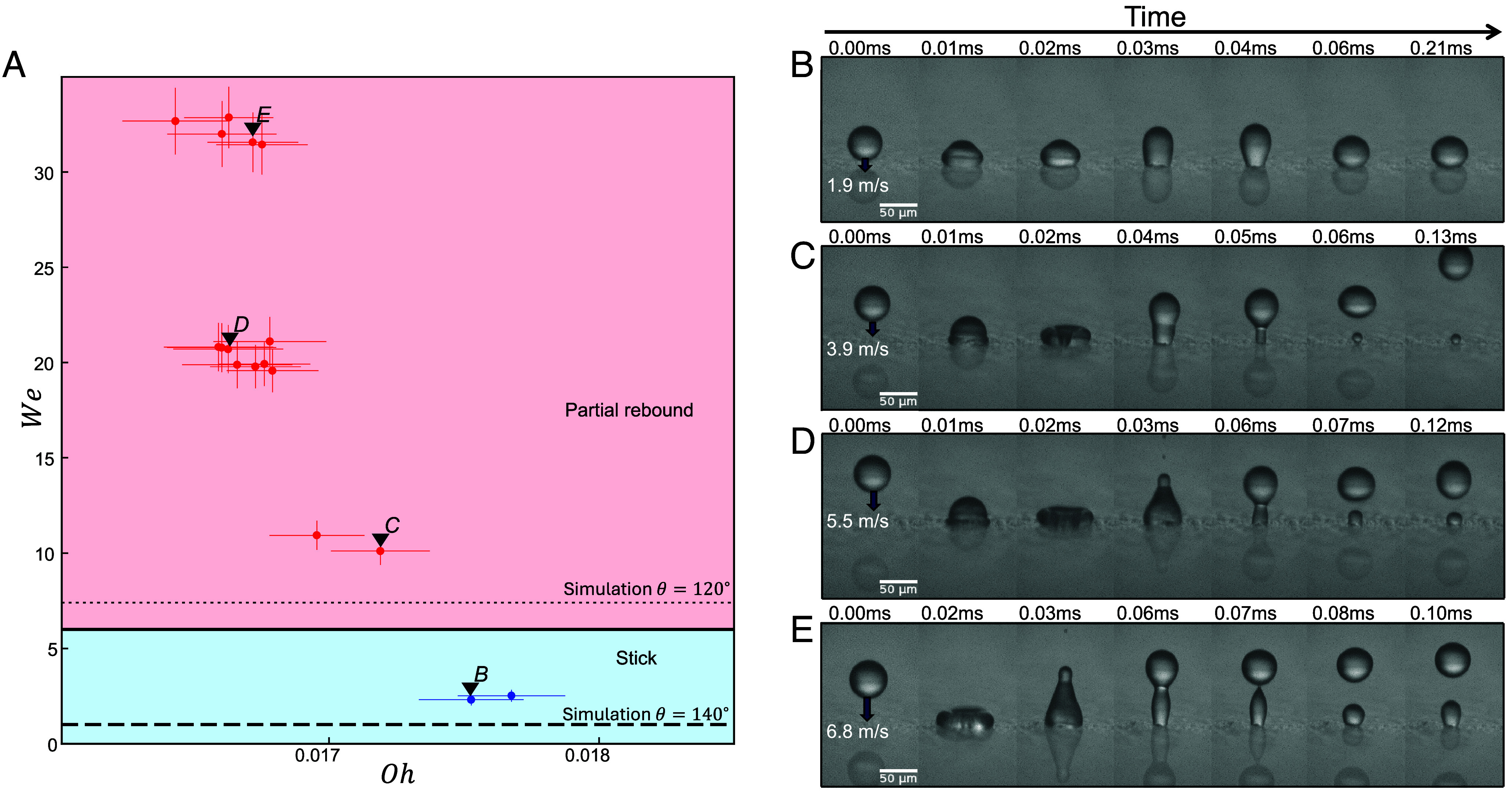
(*A*) Phase space of microdroplet impact experiments on a silicon nanosphere coated glass surface of static contact angle $\theta =140^{{}^{\circ}}$. (*B*–*E*) High-speed imaging of droplet impact outcomes: (*B*) Sticking with large oscillations, (We,Oh)=(2.31,0.0177). (*C*) Partial rebound with a small sessile droplet, (We,Oh)=(10.1,0.0172). (*D*) Partial rebound with a medium sessile droplet, (We,Oh)=(20.7,0.0166). (*E*) Partial rebound with a large sessile droplet, (We,Oh)=(31.6,0.0167).

To further test model predictions, we performed experiments on a hydrophobic surface with a higher contact angle and experimentally verified the prediction that bouncing occurs for lower We. This surface, a silicon nanoparticle-coated glass, has a static contact angle of $140^{{}^{\circ}}$. We plot the experimental results for sticking and partial rebounding in (Oh,We) phase space in Fig. 7*A*. Consistent with our intuition that weaker adhesion makes bouncing easier, for this surface, bouncing occurs at a much lower We≈10. However, when we plot the simulation transition for $\theta =140^{{}^{\circ}}$, we find it significantly underestimates We of the experimental transition. We posit the reason for this discrepancy is the greater roughness of the nanoparticle coated surfaces compared to Teflon. We measure the roughness using surface profilometry (*SI Appendix*) and find a correspondingly larger contact angle hysteresis, with a receding contact angle $\theta_{\text{r}}=120^{{}^{\circ}}$ for mm-scale water droplets. When we use this value of the receding contact angle in the numerics, we find the transition line moves to a higher value of We which is consistent with experiment, Fig. 7*A*. We then modify the numerics to introduce contact angle hysteresis and find that the droplet outcome only depends on $\theta_{\text{r}}$ and is largely independent of the advancing angle $\theta_{\text{a}}$ and the static angle θ. Although these simulations do not fully account for the effects of surface roughness in increasing the contact line dissipation and pinning, we nevertheless obtain quantitative agreement for the location of the stick-to-bounce transition. Fig. 7*B* shows the underdamped sticking that we experimentally observe in the regime where the numerics without hysteresis and with a static $\theta =140^{{}^{\circ}}$ would incorrectly predict a rebound. The fluid–solid adhesion is significantly reduced from Teflon, but the droplet still lacks enough energy to escape the surface. However, increasing the incoming speed, Fig. 7*C* shows a bouncing outcome on the nanoparticle-coated surface for droplet parameters that lead to sticking outcomes on Teflon. Notably, impacts at high We result in an additional break up of the droplet with small satellite droplets as seen at 0.03 ms in Fig. 7 *D* and *E*. These additional effects are due to higher surface roughness, but despite this the fundamental bouncing criteria still hold.

## Conclusion

In summary, we discovered that the weak surface adhesion of hydrophobic surfaces leads to a velocity-dependent stick-to-bounce transition in aqueous microdroplets. We have observed that the bouncing of microdroplets on a substrate occurs in a regime especially relevant for microdroplet processes governed by high inertia and small surface adhesion. In addition to the fundamental criterion for this transition, we have observed accompanying phenomenology such as the formation of sessile droplets during bouncing and the entrapment of air bubbles during deposition.

Significantly, we predict a universal size limit for bouncing of water-based droplets, governed by the Ohnesorge number Oh. We find that for Teflon, bouncing is completely suppressed for droplets smaller than 25 μm, and for less hydrophobic surfaces, we expect sticking for an even broader range of droplet sizes. In the context of aqueous bioaerosols, we expect that resuspension is suppressed and pathogen deposition is possible below this size limit. This observation may inform future studies on the relevant disease-transmission pathways for different droplet-size regimes.

More generally, these fundamental mechanisms have applications to natural and industrial processes where aerosols and microdroplet sprays interact with surfaces. For example, we predict that adding a hydrophilic coating will result in droplet deposition across a broad space of microdroplet parameters by suppressing the bouncing mechanism. We have identified a fundamental limit on the droplet velocity above which reliable inkjet printing on hydrophobic surfaces becomes impossible, a limit independent of the previously explored constraints imposed by the printing nozzle (8). Similarly, future 3D printing technologies that use Newtonian fluids will be affected by this speed limit, beyond which microdroplets bounce. Broadly, our research has implications across the vast application space that seeks maximum surface coverage and efficiency for industrial spray coating and crop spraying.

## Materials and Methods

### Experiments.

Microdroplet surface impact experiments were carried out using a MicroFab MJ-APB-01 30 μm piezoelectric droplet dispenser, which dispenses droplets onto a prepared substrate attached to a movable surface motor. A Photron FASTCAM NOVA S6, set at 100,000 frames per second (FPS), is used to image these droplets. This camera is attached to a Navitar Resolv4K zoom lens with a MoticPlan APO 20× objective and is backlit by a GSVitec MultiLED; see ref. 50.

For the initial substrates, glass microscope slides are cleaned with alcohol before a sheet of polytetrafluoroethylene (PTFE, i.e., Teflon) is placed on the glass. The hydrophobic Teflon surface is measured to have a static contact angle of $108^{{}^{\circ}}\pm 2^{{}^{\circ}}$ with water, as verified by imaging sessile drops at the millimeter and micron scales. Full details of surface roughness and contact angles are provided in *SI Appendix*.

Nanoparticle-coated surfaces were created by spraying SOFT99 Glaco Mirror Coat Zero onto cleaned glass slides for 5 s, creating a surface coated in silicon nanospheres. The average roughness of bare glass at 40 μm is measured to be 0.5 nm, and with the Teflon sheet, it is 6 nm. The glass with the spray coat has a roughness of 30 nm at 30 μm.

In the impact experiments, we varied droplet impact velocity, viscosity, and size, affecting the We and Oh numbers. Impact velocity was adjusted by changing the height of the droplet generator relative to the substrate. Upon generation, preimpact droplets oscillate and dampen within the first 0.5 ms, reaching a stable spherical morphology. A minimum impact height is necessary to prevent these oscillations from affecting the impact dynamics; this was chosen as the distance of travel (DOT) during 0.5 ms. Increasing the height beyond this minimum resulted in a decrease in impact velocity due to drag—an inverted height–velocity relationship compared to a freely falling droplet. The input voltage on the piezoelectric generator controlled the droplet size, resulting in two size modes of approximately 30 and 50 μm, with the latter being the most common size for our impacts. Droplet velocities and sizes are analyzed using image analysis code, allowing measurement of the impact We and Oh numbers. Uncertainties in We and Oh are propagated from the spatial and temporal resolution limits.

Deionized water was initially used for impacts. As laboratory temperature varies daily, the fluid is left to reach equilibrium before use, after which its temperature is measured to ensure room temperature. The fluid and room temperature ranged from 20 to 26 °C, leading to variations in dynamic viscosity from 1.00 to 0.89 mPas. For some runs, 5% v/v glycerol or 2.5% w/w CaCl_2_ is added to increase the viscosity to up to 1.11 mPas, expanding the *Oh* range. These changes in viscosity alter the surface tension by less than 2%, a variation deemed negligible in terms of contact angle changes. Relative humidity (RH) is measured and maintained within 10% across all runs. The droplets carry a slight electric charge due to the generation process. Additional static charging occurs between the droplet and the Teflon surface; see *SI Appendix*. The bounce threshold is not affected, as electrostatic forces are weaker than contact line friction for microdroplets and cannot significantly influence the outcome during the short contact time. However, electrostatics can influence the postbounce dynamics because their effects persist on timescales longer than the contact duration (68).

Impacts at a DOT of 1 ms and 20 ^°^C result in droplets spreading, oscillating, and sticking to the surface. Experiments are then conducted down to 0.5 ms DOT and up to an environmental temperature of 26 ^°^C. The secondary surface experiments followed the same methodology, using only water at 20 °C. Control impacts were also conducted on glass, which resulted in droplet deposition outcomes only.

### Simulations.

We make use of a phase-field method to model the two-phase droplet system. This approach introduces a phase-field variable, ϕ, which distinguishes between the liquid (ϕ=1) and the gas (ϕ=−1) phases. The phases are initialized as in Fig. 2. The Cahn–Hilliard equation governs the time evolution of the phase field ϕ, which ensures a smooth evolution of the interfaces in the system:

[12] $$\begin{array}{c} \frac{\partial \phi }{\partial t}+u\cdot \nabla \phi =\nabla \cdot \chi \lambda \nabla \psi . \end{array}$$

Here, u represents the velocity field and *χ* the interface mobility tuning parameter set as 1 m s^−1^kg^−1^. The helper function, *ψ*, is defined as

[13] $$\begin{array}{c} \psi =-\nabla \cdot \left(\epsilon_{\text{pf}}^{2}\nabla \phi \right)+\phi^{2}-1\phi , \end{array}$$

where *ϵ*_pf_ is the interface thickness, which we define by the max and min mesh elements $h_{\text{max}}$ and $h_{\text{max}}$:

[14] $$\begin{array}{c} \epsilon_{\text{pf}}=\left\{\begin{array}{cc} h_{\text{max}}, & \text{if}\;h_{\text{max}}>1.3\cdot h_{\text{min}}\\ 2\cdot h_{\text{max}}, & \text{if}\;h_{\text{max}}\leq 1.3\cdot h_{\text{min}} \end{array}\right. \end{array}$$

The parameter λ is defined to be

[15] $$\begin{array}{c} \lambda =\frac{3\epsilon_{\text{pf}}\gamma }{\sqrt{8}}, \end{array}$$

where γ is the surface tension of the fluid-air system. This formulation accurately tracks the droplet–air interface and ensures a smooth transition between the liquid and gas phases.

To model the contact angle boundary condition, which describes the angle at which the liquid phase meets the solid substrate, we impose the following boundary condition:

[16] $$\begin{array}{c} n\cdot \chi \lambda \nabla \phi =0, \end{array}$$

which equation ensures that the gradient of the phase field is consistent with the mobility at the interface, and

[17] $$\begin{array}{c} n\cdot \epsilon_{\text{pf}}^{2}\nabla \phi =\epsilon_{\text{pf}}^{2}\cos\left(\theta \right)\left|\nabla \phi \right|, \end{array}$$

which enforces the correct relationship between the interface orientation and the contact angle. The effect of the phase field ϕ evolution is incorporated into the Navier–Stokes equations via the surface tension term $F_{\text{ST}}$, which couples to fluid flow:

[18] $$\begin{array}{c} F_{\text{ST}}=\frac{\lambda }{\epsilon_{\text{pf}}^{2}}\psi \nabla \phi +\left(\frac{{\left|\nabla \phi \right|}^{2}}{2}+\frac{{\left(\phi^{2}-1\right)}^{2}}{4\epsilon_{\text{pf}}^{2}}\right)\nabla \lambda -\left(\nabla \lambda \cdot \nabla \phi \right)\nabla \phi . \end{array}$$

## Supplementary Material

Appendix 01 (PDF)

Movie S1. (A) Water microdroplet impacting and sticking to a Teflon substrate. *We* = 12 and *Oh* = 0.015. (B) 5% V/V Water/glycerol microdroplet impacting and sticking to a Teflon substrate with a bubble. *We* = 2.0 and *Oh* = 0.020. (C) Water microdroplet impacting and partially rebounding off a Teflon substrate. *We* = 24 and *Oh* = 0.015. (D) Water microdroplet impacting and partially rebounding off a Teflon substrate. *We* = 40 and *Oh* = 0.017. The videos correspond to Fig 1. (C-F) in the main text. All videos were recorded at 100,000 FPS and played at 6 FPS.

Movie S2. (A) Water microdroplet impacting and sticking to a nano particle-coated substrate. We = 2.5 and Oh = 0.017. (B) Water microdroplet impacting and bouncing off nanoparticle-coated substrate. We = 10 and Oh = 0.017. The videos used compose Fig 7. (B-C) in the main text. All videos were recorded at 100,000 FPS and played at 6 FPS.

## Data Availability

All study data are included in the article and/or supporting information.
