# Supplementary material for: Bouncing microdroplets on hydrophobic surfaces
Source: Proc Natl Acad Sci U S A. 2025 Sep 4;122(36):e2507309122. doi: 10.1073/pnas.2507309122 (PMC12435232; doi:10.1073/pnas.2507309122)
Supplement: Supplementary file 1 — Appendix 01 (PDF) [file pnas.2507309122.sapp.pdf]

PNAS

Supporting Information for

Bouncing microdroplets on hydrophobic surfaces

Jamie McLauchlan et al.

Correspondence should be addressed to jmclauchlan98@gmail.com, as3474@bath.ac.uk, or as3546@cam.ac.uk

This PDF file includes:

Supporting text
Figs. S1 to S8
Tables S1 to S2
Legends for Movies S1 to S2
SI References

Other supporting materials for this manuscript include the following:

Movies S1 to S2

Supporting Information Text

1. Experimental Results

In our work we present experimental data on microdroplet impingements, demonstrating a stick-to-bounce transition dependent on the Weber (We) and Ohnesorge (Oh) numbers. In this supplementary information, we provide additional experimental results and analysis, including explicit surface characterization. We present surface roughness data and dynamic contact angle measurements for all surfaces involved. We also consider bounce contact time, showing that it scales with the inertiocapillary timescale. Furthermore, we examine spreading behavior and demonstrate that it is independent of Oh. Additionally, we analyze droplet oscillations following a sticking impact, showing that the system is underdamped. Finally, we derive a version of the coefficient of restitution relevant for partial rebounds and calculate its value across the stick-to-bounce transition.

A. Surface Roughness and Contact Angles. This section analyzes all experimental surfaces using two key measurements: surface roughness and dynamic contact angle with water. Table S1 presents Atomic Force Microscopy (AFM) measurements of surface roughness parameters—root mean square roughness (Rq), arithmetic mean roughness (Ra), and peak-to-valley roughness (Rh)—for Teflon, untreated glass, and nanoparticle-coated glass at different scales. The data shows that nanoparticle-coated glass exhibits significantly higher roughness than untreated glass and Teflon, particularly at larger scales. The Teflon surface exhibits only a slightly higher roughness than the untreated glass.

Table S1. Roughness Measurements Comparison by Surface

Substrate and Scale	Rq (nm)	Ra (nm)	Rh (nm)
Glass, 1 µm	0.491	0.322	5.61
Glass, 40 µm	1.53	0.567	97.9
Coated Glass, 10 µm	19.6	15.9	95.8
Coated Glass, 30 µm	34.5	27.8	198
Teflon, 1 µm	2.97	2.40	22.4
Teflon, 40 µm	6.31	5.01	87.5

The static contact angle between a sessile water droplet and the surface was measured both at the micron scale using the high frame rate camera and with a 1 mm droplet using an Ossila Contact Angle Goniometer. We were able to measure the advancing θ_a and receding θ_r contact angles for the mm-scale droplets, but not for the micron-scale droplets where we were limited by the camera frame rate. For the 1 mm droplet, deionised water was slowly dispensed from a needle to measure an advancing contact angle and then drawn back in to measure the receding contact angle. The results of these measurements are presented in Table S2. The Teflon surface showed consistent static contact angle values between the micron and millimeter scales. The advancing and receding contact angles for Teflon were in line with literature values with a hysteresis of approximately 19° (1).

Table S2. Contact Angle Measurements

Substrate	Droplet Size (mm)	Static Angle (°)	Advancing (°)	Receding (°)
Teflon	0.05	108 ± 2	N/A	N/A
Teflon	1.00	107 ± 2	111 ± 5	93 ± 3
Coated Glass	0.05	139 ± 5	N/A	N/A
Coated Glass	1.00	147 ± 2	163 ± 5	121 ± 4

The coated glass exhibited inconsistent static angles between the millimeter and micron scales, likely due to variations in roughness across different length scales, as shown in Table S1. A large hysteresis $\theta_\mathrm{a} - \theta_\mathrm{r}$ of 42° was measured at the millimeter scale, consistent with a rougher surface.

B. Contact Time. We measured the contact time during a bounce and compared with previous results. Ref. (2) used a spring model to derive the bounce time of a droplet to be the inertiocapillary time t_c:

$$t_c \approx \sqrt{\frac{\pi \rho D^3}{6\gamma}} \qquad [1]$$

Bounce time vs t_c is graphed for several experiments and numerical results in Fig. S1. Most experiments with bouncing occurred with droplets of 50 µm in diameter, but 30 µm did show a shorter contact time, as expected from the above expression for t_c. There was a factor of approximately two between the experimental bounce time and the inertiocapillary timescale. In simulations, we varied the fluid density to observe different impacts with different inertiocapillary times, which were all consistent with the predicted scaling. The deviations from linearity in Fig. S1 is attributed to the transition from total rebound at low densities to partial rebound at higher densities, where surface interactions increasingly influence the dynamics.

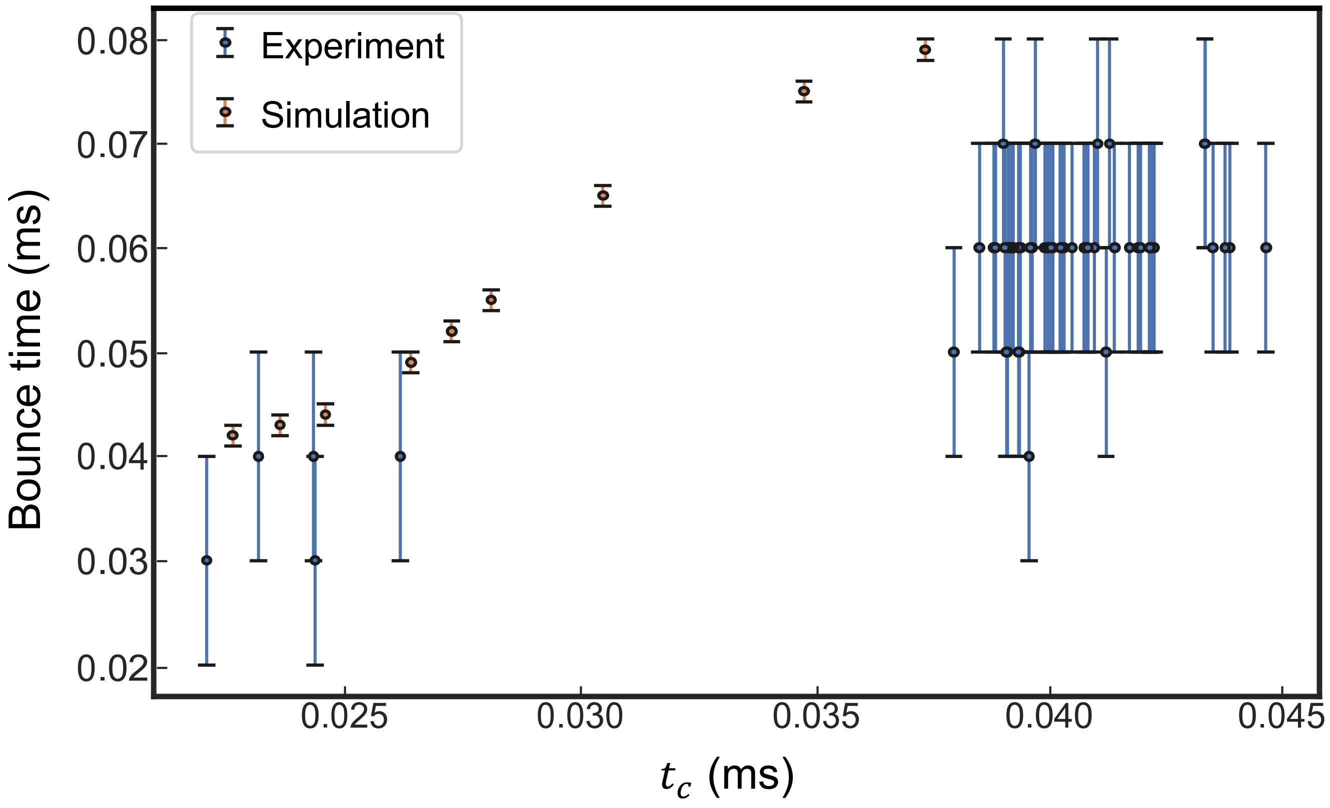

Fig. S1. Measured time for a microdroplet to bounce versus the inertiocapillary time scale, t_c, for both experimental and numerical impacts. Measured bounce time is the time the majority of a droplet is in contact with the surface, this measurement is limited by the temporal resolution of $0.01\,\mathrm{ms}$ for experiments and $0.001\,\mathrm{ms}$ for the simulations, which define the error bars. The graph shows that partial rebound events take place on approximately the same timescale as t_c.

C. Droplet Spreading. We measure the maximum spread of a droplet during impact and compare it to past literature. We find good agreement with literature values (3–5), which provides a check that any surface contamination does not affect the droplet dynamics. We take the maximum spread as the maximum horizontal distance the droplet covers on the surface.

$$\frac{D_\mathrm{m}}{D} = g(\theta)(1 + CWe^{1/2}). \qquad [2]$$

This expression was previously derived using superhydrophobic surfaces; in the hydrophobic case, we assume surface effects can be captured in the factor $g(\theta)$. This fit validates the spreading of the droplets and allows this expression of maximum spreading to be used for the energy criterion.

Fig. S2 shows a similar graph but for *Oh* at fixed *We*. The graph shows that spreading is independent of *Oh* in the low-viscosity microdroplet regime. These results confirm that the microdroplets' initial spreading behaviour is driven by inertia and counteracted by capillary forces. Hence we conclude that dissipation is minimal in the spreading phase.

D. Droplet Oscillations. We briefly analyze the oscillations in near-bouncing incidents to verify that the microdroplet system is underdamped. Upon impact in sticking events, the droplet oscillates over time. We measure this oscillation using the droplet's x and y length over time, see Fig. S3. Here, the droplet oscillations last an order of magnitude longer than the spreading process, indicating the system is underdamped. As *Oh* increases, the oscillation time decreases. The fit in Fig. S3 is a single-mode decaying sinusoidal function, which does not fully capture the multimodal oscillations which are induced by the surface. Further details of oscillations in microdroplet systems can be found in Ref. (6), which analyzes post-impact oscillations on several surface types. This is in contrast to the overdamped regime in which the droplets do not oscillate after impact. Oscillations are always seen experimentally along the stick-to-bounce boundary for microdroplets in this work, indicating this transition occurs in the underdamped regime, which highlights the role of surface adhesion in sticking.

E. Coefficient of Restitution for a Partial Rebound Event. A quantity used to measure energy dissipated in droplet bouncing is the coefficient of restitution. In this section, we discuss a form of the coefficient of restitution that also considers the change in surface energy during a partial rebound and can be used on both sides of the stick-to-bounce transition. We show an example of this for the nanoparticle coated surface.

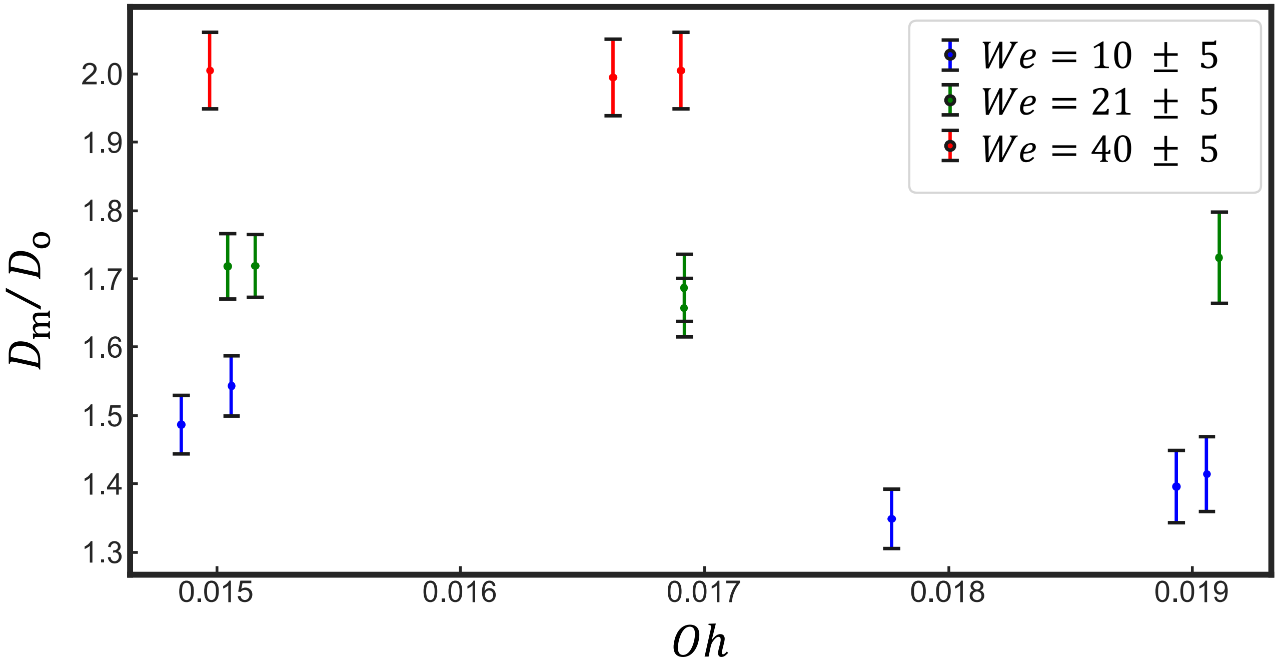

Fig. S2. Maximum spread of an impacting droplet (diameter D_{m}) divided by the original droplet diameter D_{o} versus Oh for several fixed We impacts on a Teflon surface. This graph shows that maximum spreading is independent of Oh. This graph is the companion to Fig. 5. in the main text, where we show spreading is We dependent. The data points are from the impacts of both bouncing and non-bouncing events.

The coefficient of restitution is the ratio of a droplet's final outgoing velocity (u_f) to its initial incoming velocity (u_i), which quantifies how much momentum and kinetic energy is retained:

$$\epsilon = \frac{u_f}{u_i} \qquad [3]$$

In a partial rebound, in addition to viscous loss, there is a change in the interfacial energy. To account for this, we define a ratio involving both kinetic and surface energies. This is the ratio of the final kinetic and surface energy of the outward primary droplet (D_o) and the surface energy of the sessile drop (D_s) to the initial droplet's kinetic and surface energies.

$$\epsilon' = \frac{\frac{\pi}{12}\rho D_o^3 u_f^2 + \pi D_o^2 \gamma_{FS} + \frac{\pi}{4} D_s^2(\gamma_{FS} - \gamma_{SA}) + \frac{\pi}{2} D_s^2 \gamma_{FA}(1-\cos\theta)}{\frac{\pi}{12}\rho D^3 u_i^2 + \pi D^2 \gamma_{FA}}. \qquad [4]$$

Here $\frac{\pi}{12}\rho D_o^3 u_f^2$ is the upward kinetic energy of the rebounding droplet; $\pi D_o^2 \gamma_{FS}$ is the surface energy of the rebounding droplet; $\frac{\pi}{4} D_s^2(\gamma_{FS} - \gamma_{SA})$ is the surface energy of the new fluid-solid interface for the sessile droplet minus the energy of the previous solid-air interface; $\frac{\pi}{2} D_s^2 \gamma_{FA}(1-\cos\theta)$ is the fluid-air interface for the sessile drop; $\frac{\pi}{12}\rho D^3 u_i^2$ is the initial kinetic energy of the impacting drop; and $\pi D^2 \gamma_{FA}$ is the surface energy for the impacting droplet. Writing all interfacial energies in terms of surface tension from Young's equation, and setting $\gamma_{FA} \equiv \gamma$ for clarity, we obtain:

$$\epsilon' = \frac{\frac{\pi}{12}\rho D_o^3 u_f^2 + \pi D_o^2 \gamma + \frac{\pi}{4} D_s^2 \gamma (2 - 3\cos\theta)}{\frac{\pi}{12}\rho D^3 u_i^2 + \pi D^2 \gamma}. \qquad [5]$$

Dividing through by $\pi D^2 \gamma$.

$$\epsilon' = \frac{\frac{1}{12}\frac{\rho D_o^3 u_f^2}{D^2\gamma} + \frac{D_o^2}{D^2} + \frac{1}{4}\frac{D_s^2}{D^2}(2-3\cos\theta)}{\frac{1}{12}\frac{\rho D^3 u_i^2}{D^2\gamma} + 1} \qquad [6]$$

Here we define the ratio of droplet sizes

$$R_o = D_o^2/D^2, \qquad [7]$$

$$R_s = D_s^2/D^2, \qquad [8]$$

and introduce the We number:

$$\epsilon' = \frac{\frac{1}{12} We R_o^{3/2} \frac{u_f^2}{u_i^2} + R_o + \frac{1}{4} R_s (2 - 3\cos\theta)}{\frac{1}{12} We + 1}. \qquad [9]$$

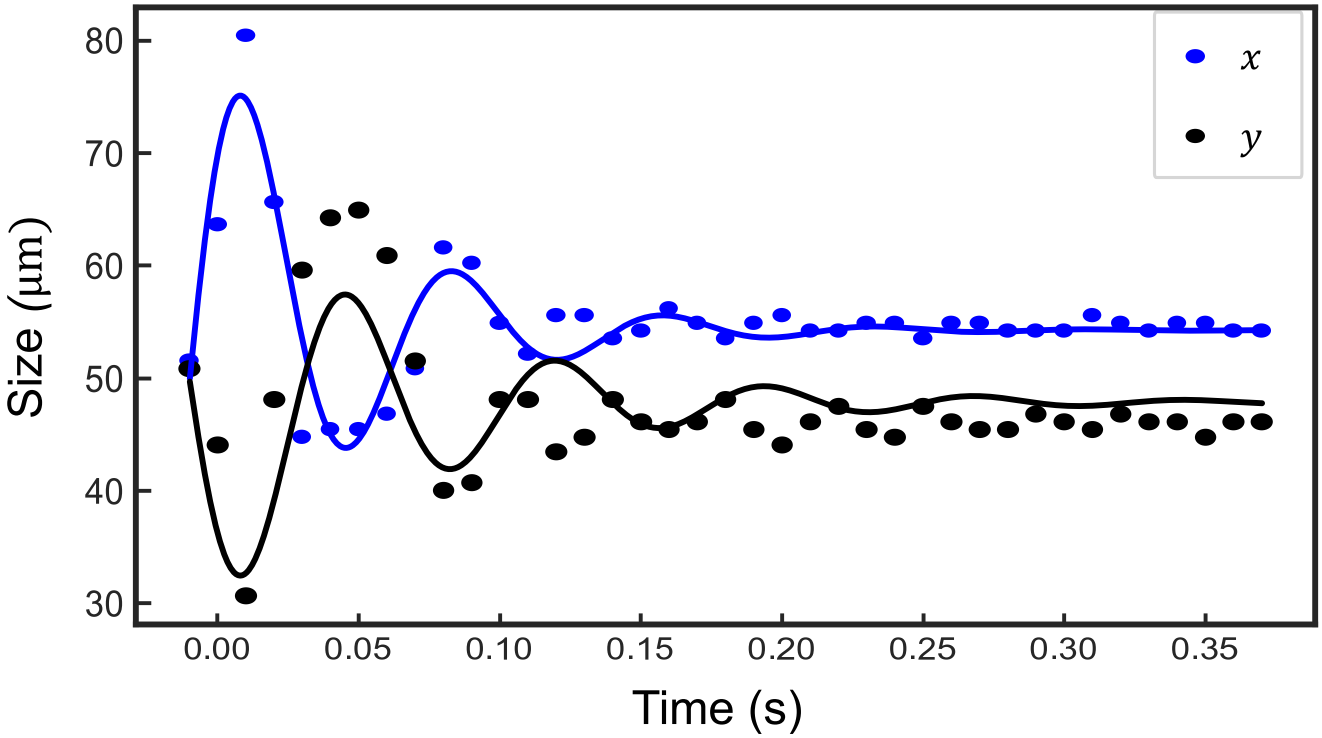

Fig. S3. Droplet oscillations graphed for a near bounce incident of (We, Oh) = (12, 0.015) on a Teflon surface. The graph shows x (height) and y (width) lengths of the droplet graphed against time. Error bars are neglected to make trends clearer. The lines represents fits for a single-mode decaying sine wave. The graph shows the underdamped nature of the sticking events we consider.

Simplifying, we find

$$\epsilon' = \frac{R_o(12 + WeR_o^{1/2}\epsilon^2) + 6R_s(1 - \frac{3}{2}\cos\theta)}{We + 12}. \quad [10]$$

This altered coefficient of restitution can be used to estimate the change in energy during a bounce event. In the case of a total rebound, $R_s = 0$ and $R_o = 1$, so that

$$\epsilon' = \frac{\epsilon^2 We + 12}{We + 12} \quad [11]$$

Which scales with usual coefficient of restitution ϵ but is not equivalent. For a sticking event, $R_o = 0$ and

$$\epsilon' = \frac{6R_s(1 - \frac{3}{2}\cos\theta)}{We + 12}. \quad [12]$$

We plot ϵ' for the nanoparticle coated surface impacts. Fig. S4 shows that as We increases, ϵ' decreases, even across the stick-to-bounce transition, highlighting a greater % of energy dissipated at higher We.

F. Droplet Charge. In our experiments, deionised water droplets acquire electrostatic charge upon contact with the Teflon substrate by donating electrons at the contact line during spreading and retraction. This contact electrification process is known to occur on hydrophobic surfaces, with some of the strongest effects observed on Teflon (7, 8). While the microdroplets do become charged, we find this has negligible influence on whether they bounce or stick. The observed stick-bounce transition aligns well with simulations that neglect electrostatics, indicating that inertial, capillary, and viscous forces dominate the dynamics.

We calculate the charge on a droplet post bounce from the re-sedimentation time. We assume the surface and droplet have equal and opposite charge, giving an estimate that microdroplets accumulate at most a few tens of femtocoulombs of charge. Although this is sufficient for electrostatic forces to exceed gravity, it does not alter the bounce threshold. However, electrostatics can influence bounce height and re-sedimentation time in near-threshold events where the rebounding droplet has limited kinetic energy. This is due to the electrostatic forces acting over a much longer time scale than viscous effects. Previous work has shown that electrostatic forces in millimetric droplets can be as significant as contact line friction (8); however, since the contact line to surface area ratio is an order of magnitude larger in micron-sized droplets, the electrostatics pre-bounce are negligible here and we neglect its full treatment. Charging effects are however important in applications where re-sedimentation must be avoided.

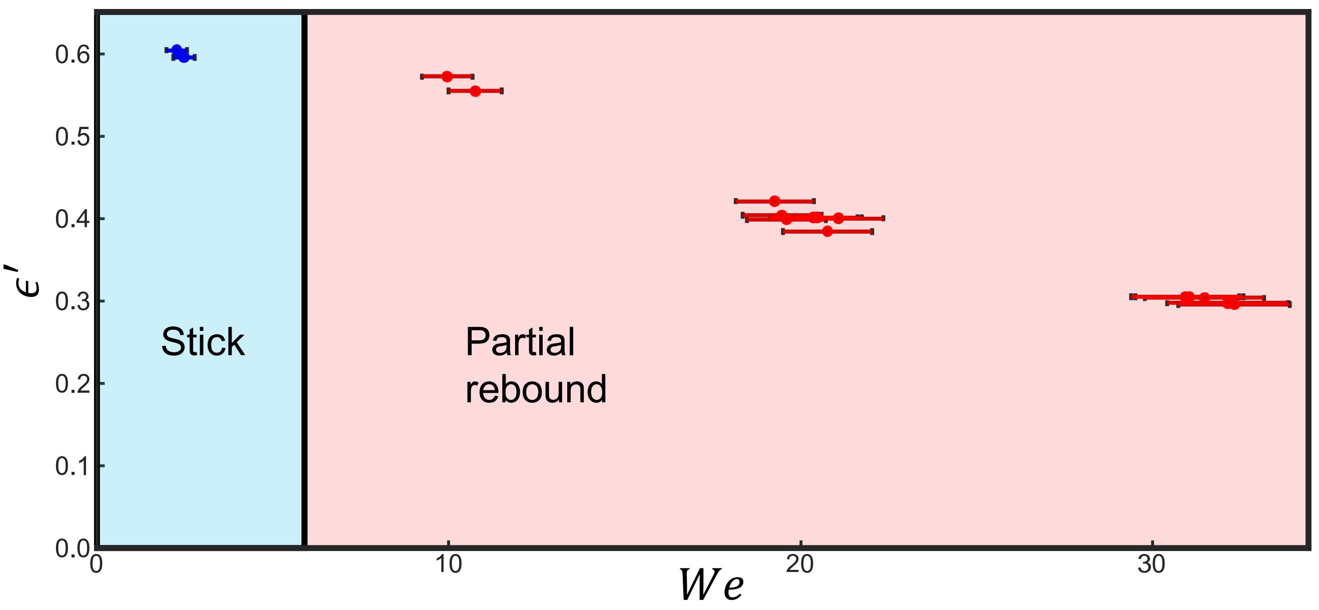

Fig. S4. Graph of the altered coefficient of restitution ϵ' vs We for all impacts on the nanoparticle coated surface. The coefficient ϵ' measures the energy remaining in the system after impact and decreases as We increases across the stick-to-bounce transition, showing greater dissipation at higher We. Error bars are from the uncertainties in size resolution and velocity.

We also observe that adding ionic surfactants or salts reduces re-sedimentation times, bringing them closer to those expected under gravity alone. We attribute this to a net positive charge imparted during piezoelectric droplet generation, which increases with ion concentration, and then neutralises upon bouncing.

2. Numerical Results

In this section, we outline further details of the simulation that allow us to make the transition plots in the main body of the paper; the numerical details are in the materials and methods section of the main text.

We carry out 120 simulations for a contact angle of 110° for various combinations of the parameters We and Oh. Although for almost all numerical simulations, we neglected hysteresis, we performed some simulations varying the advancing contact angle between 110° and 140° while keeping the receding contact angle fixed, and found that the advancing contact angle values did not alter the bouncing or sticking outcome.

As shown in the main text, increasing the We number for a set Oh numbers led to a transition of microdroplets from sticking to bouncing at sufficiently low Oh. To accurately pinpoint the transition, at fixed Oh number, we vary the We number in increasingly smaller intervals until both a sticking and a bouncing simulation took place with a difference of We numbers less than 1. We then took the transition point as the midpoint of these two simulations. All simulations for a receding contact angle of 110° are seen in Fig. S5. The lower bounds of this transition were connected by a smooth line that joins the points to give the transition line in Fig. 3(A) of the paper. The upper transition line was also included in Fig. 3(B) for completeness.

The simulation was repeated for 100 values of a contact angle of 120°, with the transition connected smoothly. Several additional simulations were performed at contact angles between 60° and 180°. Bouncing did not occur at any hydrophilic angle, and bouncing remained We-independent in the superhydrophobic limit, consistent with previous results from literature.

3. Reynolds Number Phase Diagrams

In this section, we re-express Fig. 3(A) from the main text into a Re vs Oh parameter space instead of the We vs Oh space. This rescaling provides an alternative perspective on the system since Re is a commonly used dimensionless number to describe the role of inertia. In the main text, we choose the We vs Oh space because it combines a velocity-independent variable (Oh) with one that is independent of viscosity (We), making it well suited to capture the transition dynamics.

The transition can be represented in a Re vs Oh plot, as shown in Fig. S6. The Re number is a capillary-independent composite of We and Oh, surface-tension effects are captured within Oh in this representation. However, this form is intuitively more challenging to interpret. The lowest Re at which bouncing occurs is approximately 280 for a receding angle of 110°. This corresponds to a plateau in the graph, where, at lower Oh, a higher Re indicates that for a lower viscosity, inertia dominates over viscous effects. The minimum Re required for bouncing shifts to higher Re as Oh decreases. Then, at high Oh, an even higher Re is needed for the same energy to remain in the system. The combination of viscous and inertial effects that enters Re makes the phase-space results more difficult to interpret, which is why we choose the (Oh, We) phase space for plotting results and for the energy-balance argument.

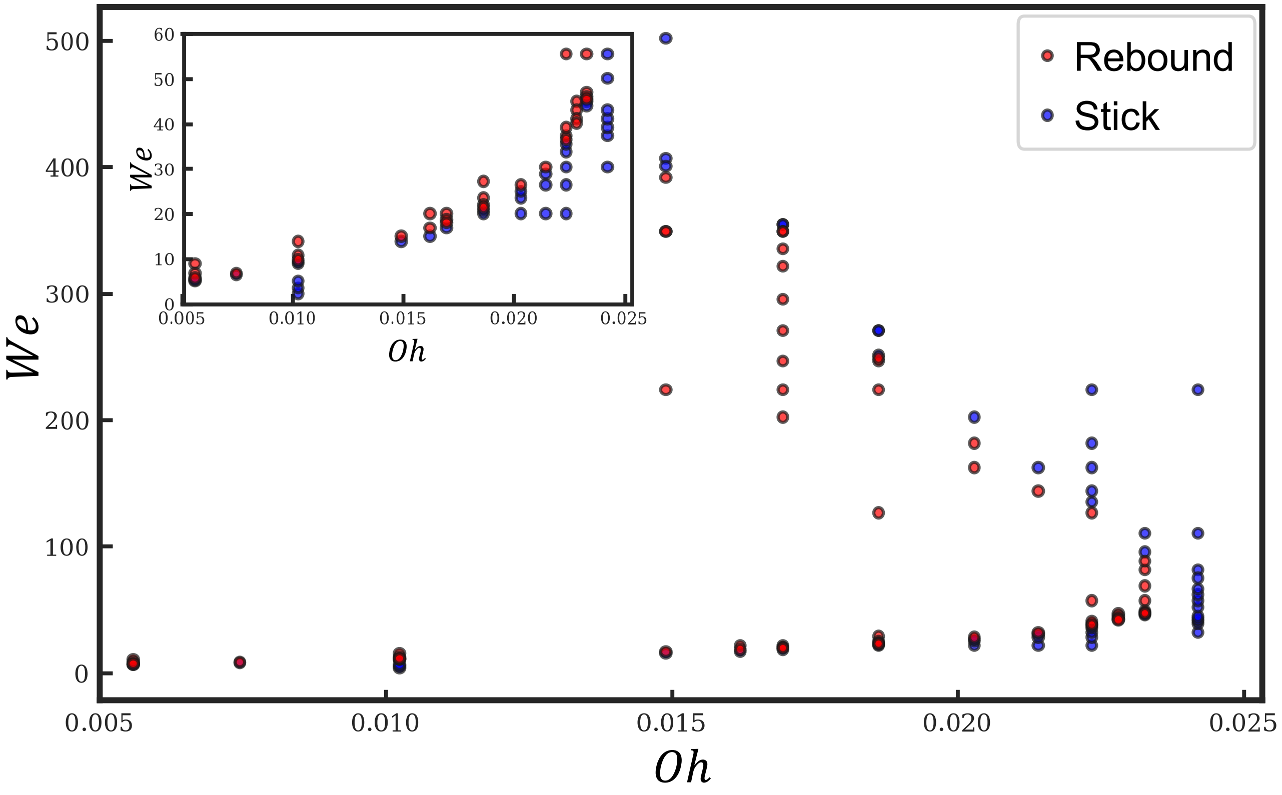

Fig. S5. All droplet simulation outcomes used to plot Fig. 2(B) in the main text. Each point corresponds to a simulation result in (We, Oh) space for a surface with a contact angle of 110°. A bounce event is defined as where most of the fluid is not in contact with the surface for at least one time-step. The simulation input values (We, Oh) are chosen to fill the parameter space as much as possible.

4. Droplet Impact Energy Considerations

In this section, we expand upon the discussion of the energy balance of a microdroplet that impacts a surface for the specific case of a partial rebound. We derive a condition to rebound based on the postulation that there must be remaining energy after an impact, expressed as

$$E_{\mathrm{k,f}} = E_{\mathrm{k,0}} - E_\gamma - E_\mu. \qquad [13]$$

To approach this derivation in more depth, we can discuss each term separately.

A. Surface Energy Term. The surface energy term in the main text E_γ is not explicitly computed. Here, we write a specific expression from the change in interfacial energy during a partial rebound. The initial droplet splits into a primary upward rebounding drop and a smaller sessile part deposited on the surface. From the conservation of volume, the sizes of these interfaces are linked:

$$D^3 = D_\mathrm{o}^3 + \frac{1}{4}D_s^3(2+\cos\theta)(1-\cos\theta)^2, \qquad [14]$$

where D_o is the rebounding droplet diameter and D_s is the spread of secondary droplet on the surface. We use the change in interfacial energy from pre- to post-impact as an approximation of E_γ, the kinetic energy converted into extra surface energy. Then,

$$E_\gamma = \pi D_o^2\gamma + \frac{\pi}{4}D_s^2\gamma_{FS} + \frac{\pi}{2}D_s^2\gamma(1-\cos\theta) - \pi D^2\gamma - \frac{\pi}{4}D_s^2\gamma_{SA}. \qquad [15]$$

This expression considers all interfaces between fluid, solid, and air and can be simplified using Young's equation linking interfacial energies to contact angle:

$$\gamma_{SA} - \gamma_{FS} = \gamma_{FA}\cos\theta = \gamma\cos\theta. \qquad [16]$$

Substituting, we find:

$$E_\gamma = \pi\gamma(D_o^2 - D^2 + \frac{1}{4}D_s^2(2-3\cos\theta)), \qquad [17]$$

which can be expressed in the general case as

$$E_\gamma = \pi\gamma D^2\left(\frac{D_o^2}{D^2} - 1 + \frac{1}{4}\frac{D_s^2}{D^2}(2-3\cos\theta)\right) \approx \pi\gamma D^2 f(\theta). \qquad [18]$$

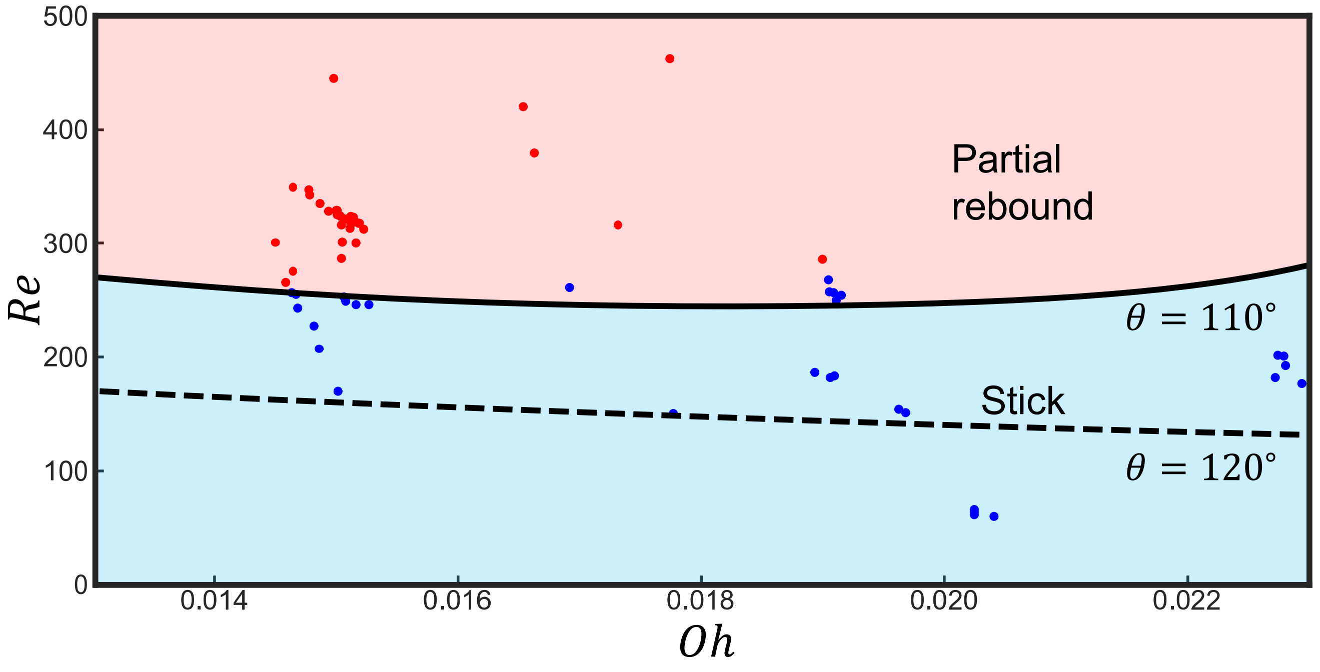

Fig. S6. A rescaling of Fig. 3(A) from the main text in terms of the Re vs Oh parameter space with both experimental points and numerical data from S5, and for a surface of contact angle of 120°. Fig. 3(A) shows a transition from bouncing to sticking at an increasing We as Oh increases. However, this transition line is not monotonic in Re vs Oh space, and the minimum Re for the transition occurs at a non-zero Oh. This non-monotonicity suggests that the transition line is simpler to model in We vs Oh parameter space. Error estimates are the same as Fig. 3(A).

This expression approximates the extra energy the interfaces now store at the point of rebounding where a fluid-solid interface is present. We use an experimental example to estimate this energy scale quantitatively. A bouncing droplet of diameter $D \approx 50\,\mu m$ is observed to have a sessile drop of diameter $D_s \approx 8\,\mu m$ on Teflon. From volume conservation this makes diameter $D_o \approx 49.8\,\mu m$, and $E_\gamma \approx 10 \times 10^{-11}$ J, meaning around 5% extra energy is stored in the interfaces. This approximation represents the energy stored in the interfaces during the point of the necking instability, where a fluid-solid interface remains.

B. Dissipation Term. In the main body of the paper, we discuss the form of the dissipated energy E_μ:

$$E_\mu = E_{\mu,3D} + E_{\mu,2D} + E_{\mu,1D}. \quad [19]$$

The bulk dissipation and boundary layer dissipation both scale as

$$E_{\mu,3D} + E_{\mu,2D} = \kappa \mu u_0^2 D t_c. \quad [20]$$

The contact time we use here is the inertiocapillary time, which we show in the main text is approximately the bounce time, that is,

$$t_c \approx \sqrt{\frac{\rho D^3}{\gamma}}, \quad [21]$$

where any constant terms are absorbed into κ, so the dissipation term can be written in terms of a viscosity-dependent part as:

$$E_\mu = \kappa \mu u_0^2 D^{5/2} \rho^{1/2} \gamma^{-1/2} + E_{\mu,1D} \quad [22]$$

The $E_{\mu,1D}$ term is from friction at the contact line as the fluid pins and depins from the surface. We write the frictional force per unit length as

$$F_f = \gamma(\cos(\theta_r) - \cos(\theta_a)) \quad [23]$$

and we write the full frictional energy loss as

$$E_{\mu,1D} = \pi \int_0^{D_m} r\gamma(\cos(\theta_r) - \cos(\theta_a))dr = \frac{\pi\gamma}{2} D_m^2 (\cos(\theta_r) - \cos(\theta_a)). \quad [24]$$

We show in the main text that in this regime, microdroplets maximum spread diameter D_m scales with the Weber number,

$$\frac{D_m}{D} = g(\theta)(1 + C We^{1/2}). \quad [25]$$

Using this scaling, we write the contact line dissipation as

$$E_{\mu,1\mathrm{D}} = D^2\gamma g^2(\theta)\Delta\cos\theta(1+CWe^{1/2})^2 = h(\theta,\Delta\theta)D^2\gamma(1+CWe^{1/2})^2. \tag{26}$$

Combining these contributions, we write the total dissipated energy as

$$E_\mu = \kappa\mu u_0^2 D^{5/2}\rho^{1/2}\gamma^{-1/2} + h(\theta,\Delta\theta)D^2\gamma(1+CWe^{1/2})^2 \tag{27}$$

C. Non-dimensionalization of Energy Balance. In this section, we rescale energy balance in terms of the Weber and Ohnesorge numbers. Using the results of the previous sections, the expression for the energy at the end of the sticking or bouncing process can be written as

$$E_{\mathrm{k,f}} = \frac{1}{2}mu_0^2 - \pi\gamma D^2 f(\theta) - \kappa\mu u_0^2 D^{5/2}\rho^{1/2}\gamma^{-1/2} - D^2\gamma h(\theta,\Delta\theta)(1+CWe^{1/2})^2. \tag{28}$$

We can take the condition for bouncing to occur when the upward kinetic energy increases just above zero,

$$\frac{\pi}{12}D^3\rho u_0^2 = \pi\gamma D^2 f(\theta) + \kappa\mu u_0^2 D^{5/2}\rho^{1/2}\gamma^{-1/2} + D^2\gamma h(\theta,\Delta\theta)(1+CWe^{1/2})^2. \tag{29}$$

Re-scaling by γD^2 non-dimensionalises the system and we obtain

$$\frac{\pi}{12}D\rho u_0^2\gamma^{-1} = \pi f(\theta) + \kappa\mu u_0^2 D^{1/2}\rho^{1/2}\gamma^{-3/2} + h(\theta,\Delta\theta)(1+CWe^{1/2})^2. \tag{30}$$

We now use the definition of the numbers We and Oh and absorb the constants in this expression to obtain:

$$We = f(\theta) + \kappa\, We\, Oh + h(\theta,\Delta\theta)(1 + 2CWe^{1/2} + C^2 We) \tag{31}$$

or

$$We = \frac{f(\theta) + h(\theta,\Delta\theta)(1+2CWe^{1/2})}{1-\kappa Oh - C^2h(\theta,\Delta\theta)}. \tag{32}$$

Expression 32 is written in terms of We and Oh as they directly encode the inertia and dissipation in the system. The expression can similarly be expressed using the Reynolds number instead of either We or Oh, with $Re = We^{1/2}Oh^{-1}$. Using this substitution we find,

$$Re^2 = \frac{f(\theta) + h(\theta,\Delta\theta)(1+2C\,Oh Re)}{Oh^2(1-\kappa Oh - C^2h(\theta,\Delta\theta))}, \tag{33}$$

see Fig. S6 for the corresponding plot of the numerical and simulation data.

5. Theoretical Phase Space

From expression 32 , we obtain the limit $\kappa Oh + C^2h(\theta,\Delta\theta) = 1$, or assuming $C^2h(\theta,\Delta\theta)$ is small, $\kappa Oh = 1$. For higher values of Oh, bouncing does not occur due high dissipation, independent of We. This heuristic approach has allowed for a simplified expression the stick-to-bounce transition parameters. This expression fits well with our numerical data when the hysteresis terms are neglected and displays linear behaviour at small Oh, in agreement with experimental and numerical observation.

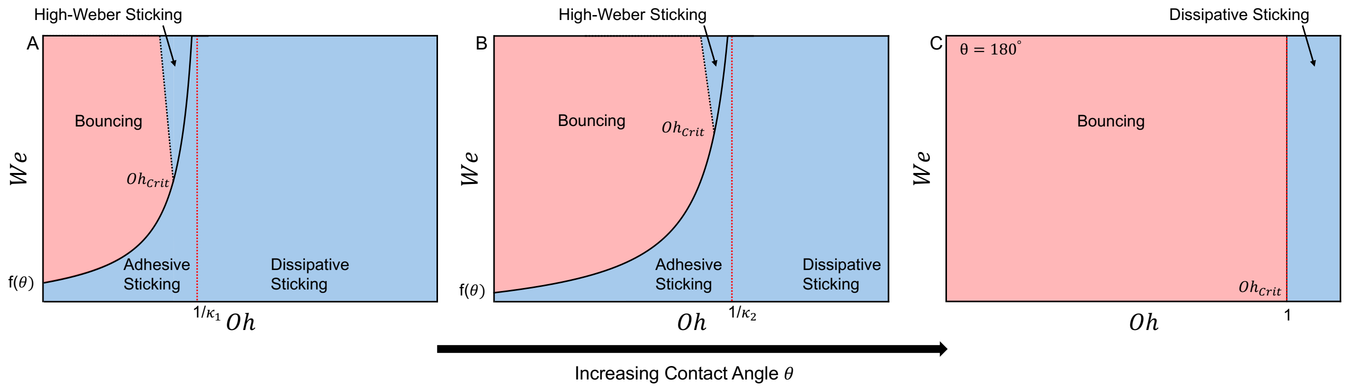

Fig. S7. Theoretical and numerical combined phase diagrams for droplet impact dynamics. (A) Moderately hydrophobic surface showing a small bouncing regime. (B) Increased contact angle expands the bouncing regime. (C) Superhydrophobic case ($\theta = 180°$), where bouncing is limited only by viscosity.

We incorporate the expression $We = \frac{f(\theta)}{1-\kappa Oh}$ into our numerical analysis to determine the lower boundary of the bouncing regime, as illustrated in Fig. S7(A–B). This boundary reveals a minimum We below which bouncing no longer occurs, and particles instead stick. We interpret this low-We sticking as being driven by adhesion, which we refer to as adhesive sticking.

As Oh increases, the dominant mechanism shifts from adhesion to dissipation, and when Oh approaches κ^{-1}, the system enters a dissipative sticking regime where energy losses due to viscosity suppress bouncing.

To provide a comprehensive picture, we also account for the high-We sticking regime observed in simulations, which lies outside the scope of the simplified theoretical prediction. As the contact angle θ increases further, the lower boundary of the bouncing regime decreases, since $f(\theta) \to 0$. This leads to the superhydrophobic limit, where bouncing becomes independent of We, consistent with observations in (9). This behaviour underscores the fundamental principle that We-independent bouncing emerges in the non-wetting limit.

6. Analytical Ball-and-Spring Model

In this section, we present details of the ball-and-spring model, including dimensionless rescaling to make contact with experiments and the estimation of the model parameters.

A. Model Set-up. The ball-spring model shows the physical mechanism behind the transition from sticking to bouncing using a simple mechanical argument. Spring models have previously been used to understand droplet bouncing and contact time (2, 10) for superhydrophobic surfaces. We build on these previous results by introducing the droplet-surface contact energy into the model.

The model consists of two masses, two springs, and a viscous damper. The two masses (M_1 and M_2) are connected to a spring (k_1) that represents surface tension and a damper (μ) that represents the viscous dissipation in the system. The second mass is connected to a spring (k_2) that represents fluid-surface adhesion. In the model, spring 1 extends beyond a critical extension, the spring breaks, the system rebounds, and mass M_1 escapes. This is representative of a necking instability. This simplified model reproduces the fundamental behaviour of the system in the bouncing and sticking regimes.

We use the following mapping between the ball-and-spring model parameters (on the left) and the fluid dynamic parameters (on the right):

$$k_1 = \zeta\gamma \quad [34]$$

$$k_2 = f(\theta)k_1 = (1+\cos\theta)\zeta\gamma \quad [35]$$

$$\mu = \alpha\eta D \quad [36]$$

$$M_1 = \frac{\pi}{6}\rho D_o^3 \quad [37]$$

$$M_2 = \frac{\pi}{6}\rho D_s^3 \quad [38]$$

Here, we introduce dimensionless scale factors ζ and α. The scale factor ζ is the scale factor between the spring constant and the surface tension. This factor can be computed from the ratio of the spring and droplet oscillation frequencies. The scale factor α is the damping scaling between the spring system and the droplet. Although a linear damper is a simplified model of viscous dissipation in a drop, it can be a valid approximation in a small range of parameter values. We also expect α to be surface dependent, with a larger spread and hysteresis corresponding to a larger dissipation. In the parameter estimation section, we estimate α and ζ for a Teflon surface in the microdroplet regime.

The equations of motion of the two masses can then be expressed as:

$$M_1\ddot{x}_1 = -k_1(x_1 - x_2) - \mu(\dot{x}_1 - \dot{x}_2), \quad [39]$$

and

$$M_2\ddot{x}_2 = k_1(x_1 - x_2) + \mu(\dot{x}_1 - \dot{x}_2) - k_2 x_2. \quad [40]$$

We then re-express these equations using droplet parameters:

$$\frac{\pi}{6}\rho D_o^3\ddot{x}_1 = -\zeta\gamma(x_1 - x_2) - \alpha\eta D(\dot{x}_1 - \dot{x}_2) \quad [41]$$

and

$$\frac{\pi}{6}\rho D_s^3\ddot{x}_2 = \zeta\gamma(x_1 - x_2) + \alpha\eta D(\dot{x}_1 - \dot{x}_2) - (1+\cos\theta)\zeta\gamma x_2. \quad [42]$$

We then non-dimensionalize this system of equations, first by introducing the dimensionless ratios of diameters:

$$m_1 = \frac{\pi}{6}\frac{D_o^3}{D^3}, \quad [43]$$

$$m_2 = \frac{\pi}{6}\frac{D_s^3}{D^3} \quad [44]$$

Then, we rescale:

$$\tilde{x}_{1,2} = \frac{x_{1,2}}{D} \quad [45]$$

$$\tilde{t} = t\frac{u}{D} \quad [46]$$

$$\dot{\tilde{x}}_{1,2} = \frac{\dot{x}_{1,2}}{u} \quad [47]$$

$$\ddot{\tilde{x}}_{1,2} = \frac{\ddot{x}_{1,2} D}{u^2} \quad [48]$$

The equations of motion are then written using dimensionless variables:

$$m_1 \rho D^2 u^2 \ddot{\tilde{x}}_1 = -\zeta\gamma D(\tilde{x}_1 - \tilde{x}_2) - \alpha\eta D u(\dot{\tilde{x}}_1 - \dot{\tilde{x}}_2) \quad [49]$$

and

$$m_2 \rho D^2 u^2 \ddot{\tilde{x}}_2 = \zeta\gamma D(\tilde{x}_1 - \tilde{x}_2) + \alpha\eta D u(\dot{\tilde{x}}_1 - \dot{\tilde{x}}_2) - (1+\cos\theta)\zeta\gamma D\tilde{x}_2. \quad [50]$$

We simplify the equations by dividing by $\rho D^2 u^2$:

$$m_1 \ddot{\tilde{x}}_1 = -\zeta\frac{\gamma}{\rho D u^2}(\tilde{x}_1 - \tilde{x}_2) - \alpha\frac{\eta}{\rho D v}(\dot{\tilde{x}}_1 - \dot{\tilde{x}}_2) \quad [51]$$

and

$$m_2 \ddot{\tilde{x}}_2 = \zeta\frac{\gamma}{\rho D u^2}(\tilde{x}_1 - \tilde{x}_2) + \alpha\frac{\eta}{\rho D v}(\dot{\tilde{x}}_1 - \dot{\tilde{x}}_2) - (1+\cos\theta)\zeta\frac{\gamma}{\rho D u^2}\tilde{x}_2. \quad [52]$$

Here, we introduce the Weber and Ohnesorge numbers to allow a direct link with the droplet system.

$$m_1 \ddot{\tilde{x}}_1 = -\zeta\frac{1}{We}(\tilde{x}_1 - \tilde{x}_2) - \alpha\frac{Oh}{\sqrt{We}}(\dot{\tilde{x}}_1 - \dot{\tilde{x}}_2) \quad [53]$$

and

$$m_2 \ddot{\tilde{x}}_2 = \zeta\frac{1}{We}(\tilde{x}_1 - \tilde{x}_2) + \alpha\frac{Oh}{\sqrt{We}}(\dot{\tilde{x}}_1 - \dot{\tilde{x}}_2) - (1+\cos\theta)\zeta\frac{1}{We}\tilde{x}_2. \quad [54]$$

For convenience, we introduce the extension of spring 1: $\Delta x = \tilde{x}_1 - \tilde{x}_2$ and arrive at the form presented in the main text:

$$m_1 \ddot{\tilde{x}}_1 = -\zeta\frac{1}{We}\Delta x - \alpha\frac{Oh}{\sqrt{We}}\Delta\dot{x} \quad [55]$$

and

$$m_2 \ddot{\tilde{x}}_2 = \zeta\frac{1}{We}\Delta x + \alpha\frac{Oh}{\sqrt{We}}\Delta\dot{x} - (1+\cos\theta)\zeta\frac{1}{We}\tilde{x}_2 \quad [56]$$

The system's initial condition is that it starts at its equilibrium and moves downwards, equivalent to the droplet spreading and energy being stored in the interfaces. This downward velocity is encoded in the system's We number, such that the dimensionless velocity is unity:

$$\tilde{x}_1 = \tilde{x}_2 = 0 \quad [57]$$

and

$$\dot{\tilde{x}}_1 = \dot{\tilde{x}}_2 = -1 \quad [58]$$

We solve this system for combinations of We, Oh, and θ for given parameter values. We state a bounce occurs when for spring 1, Δx exceeds a critical length scale. We set this lengthscale to be $\Delta x = 1$ in the non-dimensionalized units.

B. Parameter estimation. This section estimates the dimensionless scaling factors between the ball-and-spring model and the microdroplet system.

The value of $\zeta = 1$ matches the experimentally observed oscillation frequencies. To estimate α, we use two independent methods. First, we compare the damping of oscillation in a microdroplet impact (i.e. Fig. S3) to the damping of the spring system and the coefficient of restitution to the damping for a bouncing microdroplet. We observe that for an impact with a Teflon surface, microdroplets lose between 30 and 50% of their initial energy, depending on the numbers We and Oh. This compares to 1% in the model with the same Oh and an α of 1. Setting α to 50 matches the energy dissipation approximately for the range of Oh 0.015 to 0.025, which corresponds to our experiments. Note that this estimate is only valid for the specific surface used, which is Teflon. Indicating additional dissipative mechanisms makes dissipation approximately 50 times greater than the linear bulk.

The second estimate of α uses the energy dissipated in a microdroplet. In the main text, we say the dissipation is given by:

$$E_\mu = E_{\mu,\mathrm{3D}} + E_{\mu,\mathrm{2D}} + E_{\mu,\mathrm{1D}} \quad [59]$$

where $E_{\mu,\mathrm{3D}}$ for microdroplets impacting between 1 and 10 m/s is between 1×10^{-12} J to 1×10^{-10} J. The value $E_{\mu,\mathrm{2D}}$ is approximately an order of magnitude larger, scaling as $Oh^{1/2}$. We also estimate the contact line dissipation using Ref. (11):

$$E_{\mu,\mathrm{1D}} = \frac{\pi\gamma}{2} D_\mathrm{m}^2 \left(\cos(\theta_r) - \cos(\theta_a)\right). \quad [60]$$

Contact angle hysteresis for Teflon is measured to be approximately 19°, and the maximum spread varies from 40 to 90 µm for the range of We numbers we consider. This gives a value of $E_{\mu,\mathrm{1D}}$ approximately 3 to 6 × greater than $E_{\mu,\mathrm{3D}}$.

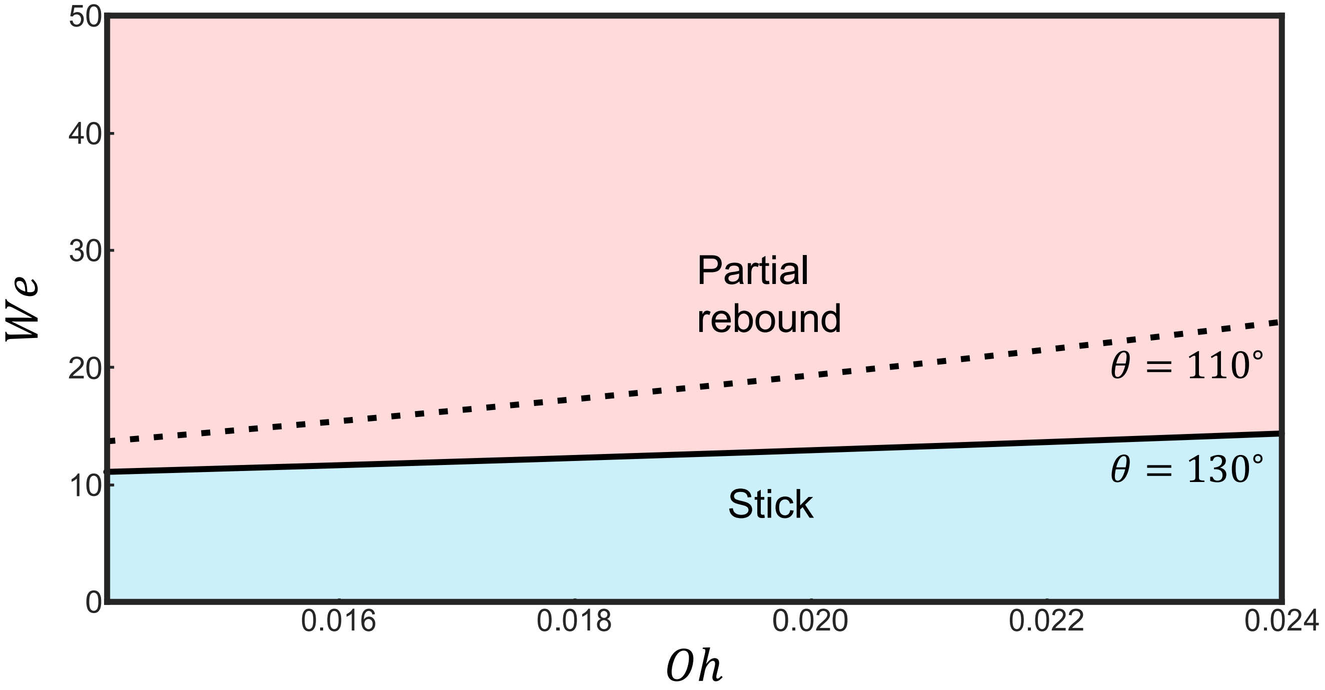

Fig. S8. Phase space of sticking vs bouncing based on the ball-and-spring model for different sets of parameters. Here the two lines correspond to $(\alpha, \theta) = (30, 130^\circ)$ (solid) and $(\alpha, \theta) = (50, 110^\circ)$ (dashed), with this second line corresponding to the line plots in Fig. 6(c) of the main text.

Combining these approximations suggests a scaling of between 30 and 60 for α. The value $\alpha = 50$ aligns well with both of these estimation methods. The α value increases with hysteresis and hydrophilicity, so estimates must be adjusted for a relevant surface.

This model predicts the stick-to-bounce transition for the parameters we considered. Fig. S8 shows that the ball-and-spring model replicates some of the physics of varying the contact angle in experiments. For example, a droplet bounces on a more hydrophobic surface at a lower value of *We*.

Movie S1. (A) Water microdroplet impacting and sticking to a Teflon substrate. $We = 12$ **and** $Oh = 0.015$**. (B) 5% V/V Water/glycerol microdroplet impacting and sticking to a Teflon substrate with a bubble.** $We = 2.0$ **and** $Oh = 0.020$**. (C) Water microdroplet impacting and partially rebounding off a Teflon substrate.** $We = 24$ **and** $Oh = 0.015$**. (D) Water microdroplet impacting and partially rebounding off a Teflon substrate.** $We = 40$ **and** $Oh = 0.017$**. The videos correspond to Fig 1. (C-F) in the main text. All videos were recorded at 100,000 FPS and played at 6 FPS.**

Movie S2. (A) Water microdroplet impacting and sticking to a nano particle-coated substrate. $We = 2.5$ **and** $Oh = 0.017$**. (B) Water microdroplet impacting and bouncing off nanoparticle-coated substrate.** $We = 10$ **and** $Oh = 0.017$**. The videos used compose Fig 7. (B-C) in the main text. All videos were recorded at 100,000 FPS and played at 6 FPS.**

References

1. H Kamusewitz, W Possart, The static contact angle hysteresis obtained by different experiments for the system ptfe/water. *Int. J. Adhesion Adhesives* **5**, 211–215 (1985).
2. K Okumura, F Chevy, D Richard, D Quéré, C Clanet, Water spring: A model for bouncing drops. *Europhys. Lett.* **62**, 237–243 (2003).
3. E Villermaux, B Bossa, Drop fragmentation on impact. *J. Fluid Mech.* **668**, 412–435 (2011).
4. N Laan, KG de Bruin, D Bartolo, C Josserand, D Bonn, Maximum diameter of impacting liquid droplets. *Phys. Rev. Appl.* **2**, 044018 (2014).
5. V Sanjay, D Lohse, Unifying theory of scaling in drop impact: Forces and maximum spreading diameter. *Phys. Rev. Lett.* **134**, 104003 (2025).
6. LP McCarthy, JP Reid, JS Walker, High frame-rate imaging of the shape oscillations and spreading dynamics of picolitre droplets impacting on a surface. *Phys. Fluids* **35**, 122010 (2023).

7. D Díaz, et al., Charging of drops impacting onto superhydrophobic surfaces. *Soft Matter* **18**, 1628–1636 (2022).
8. D Díaz, et al., Self-generated electrostatic forces of drops rebounding from hydrophobic surfaces. *Phys. Fluids* **35**, 017111 (2023).
9. V Sanjay, P Chantelot, D Lohse, When does an impacting drop stop bouncing? *J. Fluid Mech.* **958** (2023).
10. A Jha, P Chantelot, C Clanet, D Quéré, Viscous bouncing. *Soft Matter* **16**, 7270–7273 (2020).
11. D Quéré, Wetting and roughness. *Annu. Rev. Mater. Res.* **38**, 71–99 (2008).
